# Beneficial microbes in plant salt stress tolerance: mechanisms, antioxidant defense, and multi-omics insights

**DOI:** 10.3389/fmicb.2026.1965097

**Published:** 2026-10-26

**Authors:** Shubhangi Shree, Anjali Chaudhary, Subhash Kumar, Kunal Singh, Namo Dubey

**Affiliations:** 1 School of Bio Sciences and Technology, Vellore Institute of Technology, Vellore, India; 2 CSIR-Institute of Himalayan Bioresource Technology, Palampur, India; 3 Academy of Scientific and Innovative Research (AcSIR), Ghaziabad, India

**Keywords:** antioxidant molecules, enzymatic defense, multi-omics approaches, plant-microbe interactions, salinity stress

## Abstract

Salinity stress represents a significant environmental challenge that affects plant growth, resulting in adverse impacts on crop productivity and overall yield. Salt accumulation in plants affects cellular homeostasis and provokes osmotic and ionic imbalance. It stimulates higher reactive oxygen species (ROS) production leading to oxidative stress damage and eventual cellular dysfunction. Beneficial microorganisms like plant growth-promoting rhizobacteria, arbuscular mycorrhizal fungi, endophytic fungi, and rhizosphere-associated microbes have recently emerged as resources for sustainable solutions to enhance plant resiliency against salinity stress. Their effects can involve osmotic adjustment, ion sequestration, oxidative-stress mitigation, phytohormone regulation, and rhizosphere-mediated stress buffering. The mechanisms to impart such benefits may include improved nutrients acquisition, assist in osmotic regulation, modulation of phytohormones signaling, and maintenance of cellular ion homeostasis. Often, beneficial microorganisms carry the potential to trigger both enzymatic and non-enzymatic antioxidant defense mechanisms. These responses contribute to oxidative-stress mitigation, yet, their interpretation requires contemplation of fine balance between oxidative damage and plant responses. For example, selected microbial inoculation studies have reported changes in the activity of crucial antioxidant enzymes, like superoxide dismutase, ascorbate peroxidase, catalase, and glutathione reductase. However, the magnitude and the direction of these responses are context dependent. Multi-omics approaches, including amplicon and shotgun metagenomics, metatranscriptomics, host transcriptomics, proteomics, and metabolomics, have expanded our understanding of the taxa, genes, transcripts, proteins, and metabolites associated with plant–microbe interactions under salinity. Therefore, here an effort has been made to present the evidential support for reported microbial effects and their proposed mechanisms of salt tolerance, with a focus on enzymatic and non-enzymatic defense mechanisms and the learnings of multi-omics studies. Frontier integrative mechanisms, including microbiome engineering, synthetic microbial consortia, and systems biology frameworks, are discussed for cultivating climate-resilient crops and sustainable agricultural systems in the salt-affected environments.

## Introduction

Soil salinization is regarded as one of the most significant constraints and an emerging threat for agricultural production and sustainability of arable ecosystems, globally. Accumulation of soluble salts in agricultural land has occurred owing to climate change, seawater intrusion, intensive irrigation, insufficient drainage, and human-induced changes in land usage. Soil salinity currently affects approximately 1.4 billion hectares, accounting for more than 10% of the Earth’s land surface, and threatens over one billion additional hectares owing to climate change and unsustainable land management^1^. According to projections with enhanced aridity, salt-affected land may constitute 24%–32% of the total land area (see text footnote 1). Salinity stress causes negative impacts on plants growth at different biological levels. High concentrations of Na^+^ and Cl^–^ decrease soil water potential, restrict water uptake, disturb ion balance, hinder nutrient assimilation, decrease photosynthesis efficiency, cause overproduction of reactive oxygen species (ROS), which leads to protein, lipid, nucleic acid, and organelles damage (Hasanuzzaman et al., 2021; Zandi and Schnug, 2022; Rao et al., 2025). To cope with stress, plants employ diverse physiological, biochemical, and molecular mechanisms that enable adaptation and survival (Verma et al., 2024; Dubey et al., 2025). This includes intracellular control of Na^+^/K^+^ ratio via the Salt Overly Sensitive (SOS) signaling pathway, toxic ion storage in vacuoles by NHX transporters, ion transportation by HKT transporters, synthesis of compatible osmolytes such as glycine betaine, proline, and activation of antioxidant defense systems (Wang et al., 2019; Ali et al., 2021; Maach et al., 2024; Admas et al., 2025).

Salinity stress not only involves ionic toxicity but has adverse effects on many other cellular systems too. Salinity stress at first causes osmotic stress through lowering the water potential of soil, causing a limitation in water uptake and creating physiological drought conditions. When sodium ions accumulate inside the cells of plants, ionic imbalance affects nutrient absorption, enzyme function, membrane stability, and photosynthesis by competing with other essential cations like potassium and calcium (Wang et al., 2019; Ali et al., 2021). Such basic disturbances in the functioning of organisms very quickly cause metabolic malfunctioning and production of excessive amounts of ROS. Even though ROS is used as an important signaling mechanism during the development process of the organism and in sensing stress conditions. An imbalance in the cellular redox level causes peroxidation of lipids, protein damage, DNA damage, malfunctioning of chloroplasts, and ultimately plant growth arrest (Hasanuzzaman et al., 2021; Zandi and Schnug, 2022; Rao et al., 2025). To mitigate oxidative stress, plants have evolved a multi-faceted defense system that involves regulation of ions, osmoprotection, and antioxidant metabolism (Dubey et al., 2026). Equally important is the activation of antioxidant defense mechanisms that preserve cell redox balance. The antioxidant enzymatic defense includes the catalytic conversion of ROS to less harmful species performed by superoxide dismutase (SOD), ascorbate peroxidase (APX), catalase (CAT), peroxidases (POD), glutathione reductase (GR), and ascorbate-glutathione cycle enzymes (Hasanuzzaman et al., 2021; Zandi and Schnug, 2022; Dubey et al., 2025; Figure 1). Enzymatic antioxidant defense is supplemented by the action of the non-enzymatic antioxidants, such as ascorbate, glutathione, carotenoids, tocopherols, flavonoids, and phenolics that function as redox buffers, membrane stabilizers, photosynthetic apparatus protection agents, and redox signaling compounds (Hasanuzzaman et al., 2021; Rao et al., 2025; Figure 2). Overall, it is this interconnected system that decides the fate of plants when exposed to saline conditions.

**FIGURE 1:**
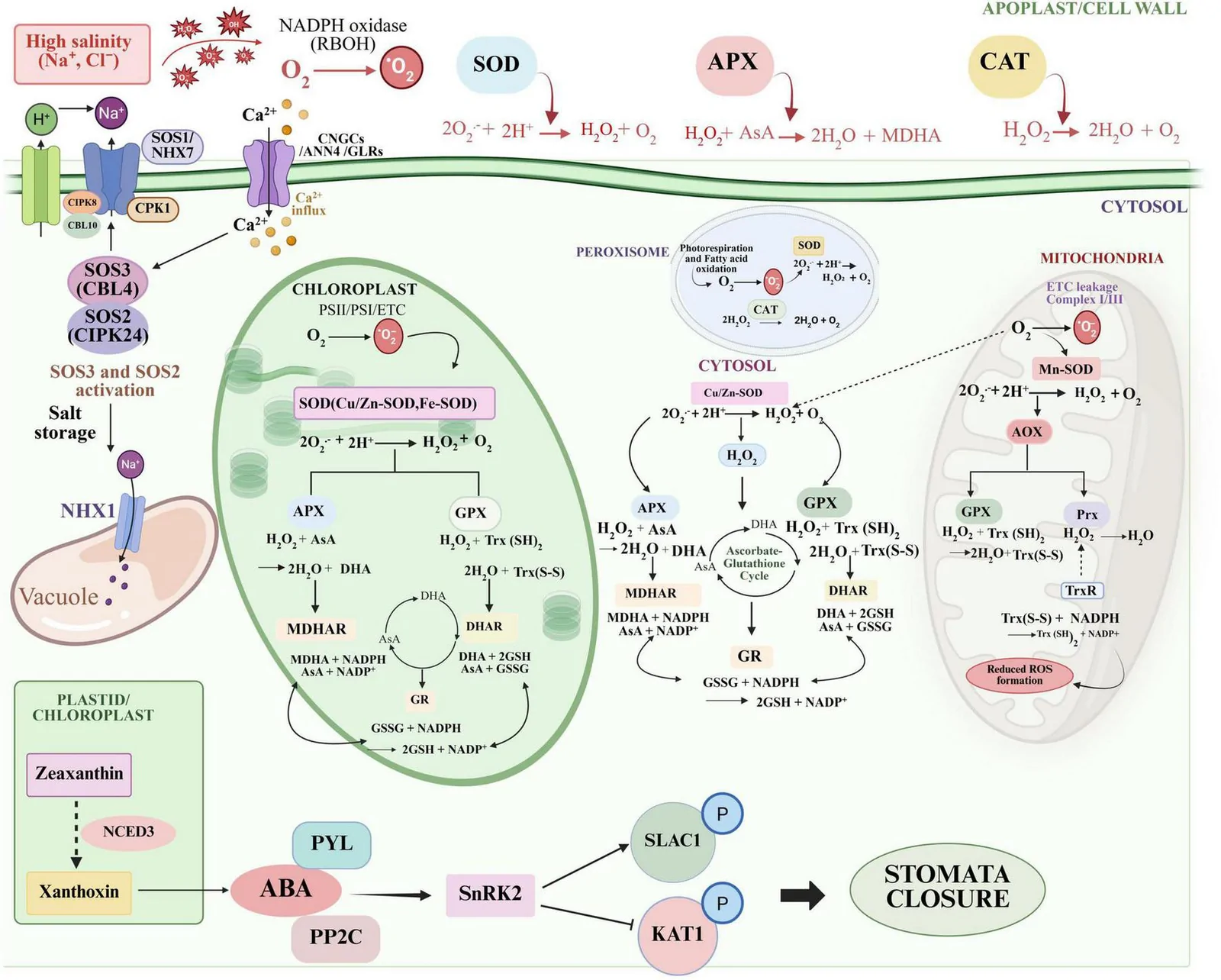
Enzymatic antioxidant defense under salinity stress. Excess ROS generated in different cellular compartments are detoxified by antioxidant enzymes, including SOD, APX, CAT, GPX, Prx, and the ascorbate–glutathione cycle (MDHAR, DHAR, and GR), together with the thioredoxin/peroxiredoxin system, to maintain cellular redox homeostasis. Figure was created using BioRender.

**FIGURE 2:**
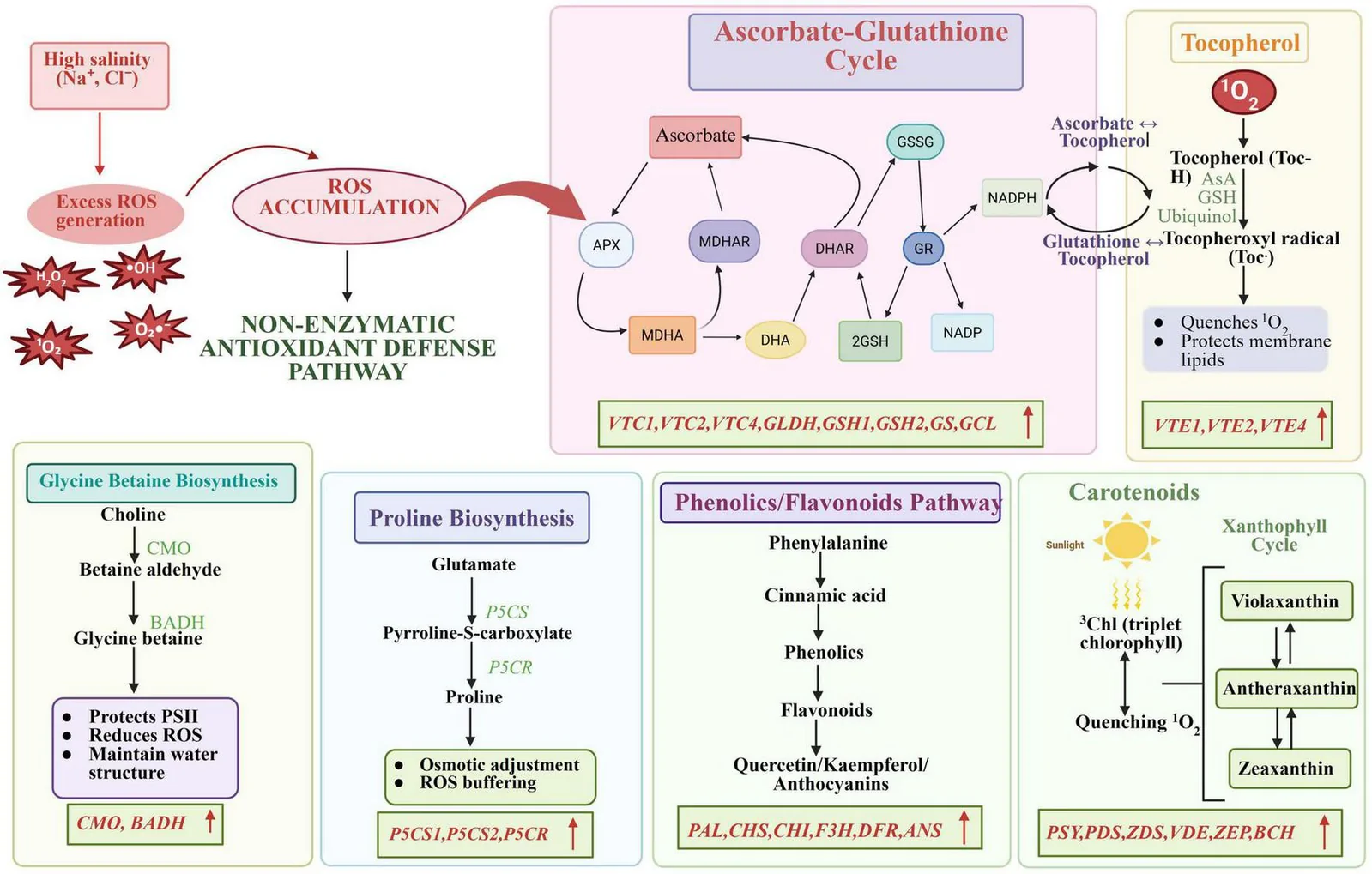
Non-enzymatic antioxidant defense in halophytes. Ascorbate, glutathione, α-tocopherol, carotenoids, phenolics, flavonoids, proline, and glycine betaine protect against oxidative damage, maintain redox balance, and enhance salinity tolerance. Salt-responsive genes involved in these pathways are indicated. ROS, reactive oxygen species; AsA, ascorbate; GSH, reduced glutathione; CMO, choline monooxygenase; BADH, betaine aldehyde dehydrogenase; P5CS, Δ^1^-pyrroline-5-carboxylate synthetase; P5CR, Δ^1^-pyrroline-5-carboxylate reductase. Figure was created using BioRender.

In the current scenario, plant microbiome has become an important factor responsible for plant adaptation to salinity stress (Ren Y. et al., 2025). Different microbes colonize the rhizosphere, endosphere, and phyllosphere and are involved in several processes including nutrient acquisition, phytohormone synthesis, stress responses, disease resistance, and environmental acclimation (Yang et al., 2023; Ren Y. et al., 2025). These microbial communities are important for providing salt tolerance in plants through direct and indirect mechanisms (Giannelli et al., 2023; Boorboori and Lackóová, 2025). Such microbes are responsible for nutrient uptake, synthesis of IAA and EPS, production of compatible solutes, induction of antioxidant responses, regulation of ethylene production via ACC deaminase activity, and modulation of ion transport in the host (Zheng et al., 2021; Giannelli et al., 2023). Salinity influences both plants and the microbial communities inhabiting their rhizosphere by exerting strong selective pressure on their composition and function. Therefore, high-salinity environments are notable for the marked changes in structure, diversity, and function of microbial communities. Taxa tolerant to osmotic stress, capable of regulating ion homeostasis and able to produce protective metabolites become enriched in high salinity. Studies of microbial community structure in mangrove ecosystems, rice rhizosphere, vineyards, and agricultural saline soils reveal major shifts due to salinity and the corresponding enrichment of salt-tolerant species (Yang et al., 2023; Wang B. et al., 2023; Wang et al., 2026; Zhou et al., 2024). For example, metagenomic comparison of rhizospheric microbial communities from salt-tolerant grapes revealed enrichment of pathways involved in sulfur metabolism, glutathione metabolism and chemotaxis, while metagenomic analysis of saline rice microbiome pointed to increased representation of genes coding Na^+^/H^+^ antiporter, V/A-type ATPase, glycine betaine synthesis and proline pathway (Wang B. et al., 2023; Wang et al., 2026). Furthermore, investigations of mangroves and rice rhizospheres revealed salinity-dependent shifts in microbial community composition and microbial ecological functions such as nutrient cycles, phototrophy, and oxidant metabolism (Yang et al., 2023; Zhou et al., 2024). These studies indicated that plant survival in saline environments relies on both inherent stress tolerance and the beneficial functions of associated microbial communities. Major advances in omics technologies over the last decade have allowed us to reach this goal by using information on the composition of microbial populations, their gene regulation, expression of proteins, metabolite content, and inter-kingdom signaling pathways.

Several multi-omics approaches have unraveled the complex molecular intricacies behind the mechanism of salt tolerance via microorganisms on plants. They revealed microbial roles in ion homeostasis, osmo-protection, nutrient acquisition, hormonal control, antioxidant defense, and metabolic adaptation in plants like wheat, *Metasequoia glyptostroboides*, licorice, and rose (Xiao et al., 2024; Ren et al., 2024; Qiu et al., 2026; Huang-Fu et al., 2026; Weng et al., 2025). These analyses reveal the regulatory interactions between microbial activities and the host’s physiological and metabolic processes under salt stress conditions. A few metagenomic and metatranscriptomic analysis reveal adaptive microorganisms under saline conditions. As such, microbial genes involved in ion transport, osmoprotection, energy metabolism, nutrient cycling, as well as osmoadaptation and carbon metabolism in microbes can get activated (Wang et al., 2026; Zhou et al., 2025).

Although there is an emerging trend in using high-throughput multi-omics to understand plant-microbe interactions under salinity, a key gap persists in establishing the connections between these datasets into a cross-kingdom mechanistic framework. The majority of studies are centered around investigating individual omics layers, and their relationships with microbial community structure and potential are poorly understood in terms of how they affect microbial activity, host responses, metabolism, and salt tolerance. We provide a critical synthesis of the multi-omics investigations, emphasizing the connections between molecular observations and macroscopic physiology and functionality. Instead of approaching the microbial profiles, host responses, and metabolic shifts as independent aspects, we assess how their combination contributes to the mechanistic understanding of microbiome effects on salt tolerance. First, we discuss the biphasic nature of salinity stress and its implications for plant physiology. Then, we focus on the contributions of individual microorganisms and their communities to the modulation of enzymatic and non-enzymatic antioxidant systems and cell protection. We review the results of multi-omics studies, including the changes in microbial community composition, potential, activity, host responses, and metabolism. We pay specific attention to the extent of integration in individual studies, including experimental validation and causal inference. Finally, we discuss the mechanistic challenges in translating these insights to the field and propose emerging concepts for considering the system-level properties of microbiomes in salt-affected soils to harness their potential for agriculture.

## Biphasic salt stress

Soil salinity disrupts the normal functioning of plants via a well-known biphasic process initiated by an osmotic shock accompanied by accumulation of slower ionic toxicity (Ma et al., 2026). During the initial osmotic phase, elevated NaCl concentration in the rhizosphere significantly reduces soil water absorption capacity within hours, causing cellular drying out, decrease of turgor pressure, and limits cell differentiation and growth (Rehman et al., 2025; Ren P. et al., 2025). The later ionic phase occurs due to significant accumulation of salts such as sodium (Na^+^), magnesium (Mg^2+^), sulfates (SO_4_^2–^), and chloride (Cl^–^) in plant tissue causing nutrient and ion imbalance. Ultimately, these two phases disrupt the physiological and metabolic activity of the plants (Roy and Chowdhury, 2020; Asad et al., 2022). In addition to the biphasic process, salinity also causes the production of a high amount of ROS, which further initiates cellular damage (Kesawat et al., 2023).

Together, nutrient uptake, plant water relations and cellular homeostasis are highly impaired by this interrelated biphasic stressor, making the plant more susceptible to growth inhibition and decreased yield (Rehman et al., 2025). An excessive production of ROS under a saline environment accelerates cellular destruction by causing protein oxidation, lipid peroxidation, and nucleic acid breakage (Kesawat et al., 2023). The treatment and exposure duration of salt, along with the tolerance capacity of plant species, determine the severity of this cascade (Yang and Guo, 2018). As a whole, such imbalances show a variety of physiological effects that drastically change plants molecular and biochemical balances.

The osmotic and ionic phases are not entirely sequential but are mechanistically linked. The limitations on water absorption during the osmotic phase can decrease stomatal conductance and carbon assimilation, which in turn diminishes the metabolic reserves needed to handle subsequent ionic toxicity (Ma et al., 2026). Microbiomes associated with roots affect both phases through mechanisms that overlap rather than being exclusive to each phase. In the osmotic phase, extracellular polymeric substances (EPS) produced by microbes can improve water retention in the rhizosphere (Liu et al., 2022; Rasulov et al., 2020), and microbial influences on the host’s compatible-solute metabolism aids in early osmotic adjustment (Zuzunaga-Rosas et al., 2024; İkiz et al., 2024). During the progression of the ionic phase, the role of microbes shifts to affect the host’s management of Na^+^, potentially influencing the transport of Na^+^ from roots to shoots and its storage in vacuoles, which aids in preserving the cytosolic K^+^/Na^+^ ratio (Ren P. et al., 2025). According to the interconnected nature of osmotic and ionic stress factors, these microbial roles should be viewed as complementary across both phases, rather than as distinct strategies limited to a single phase.

## Physiological consequences of salinity stress

Accumulation of excess NaCl causes vital consequences such as ion toxicity, ion imbalance, oxidative damage, and disrupted signal transport. Consequently, all these together cause impairment and imbalance in plant growth and survival by different mechanisms.

### Ion imbalance, toxicity, and transport signaling

The elevated accumulation of Na^+^ and Cl^–^ ions inside plant tissues causes ionic toxicity during salt stress (Mahmood et al., 2024). Significant metabolic dysfunction occurs as a result of Na^+^ ion accumulation in plant roots and leaves because of fierce competition for enzyme binding sites with essential cellular nutrients (such as K^+^ and Ca^2+^), causing reduced cellular activity (Ma et al., 2026). In addition, ion toxicity and increased salt levels affect photosynthetic machinery by interfering with the function of enzymes, chloroplasts, and the cell membrane. The accumulated Na^+^ ions inside leaf tissues degrade chlorophyll and inhibit chlorophyll biosynthesis, which is essential for capturing light during photosynthesis (Lu et al., 2023). On the other hand, damaged chloroplast ultrastructure have disruption in thylakoids, leading to disrupted stacking of grana, ultimately disrupting the light- dependent reactions (Aliyeva et al., 2025).

Salt stress also affects the enzymes and proteins involved in the transport of electrons during photosynthesis. During osmotic shock, plants quickly undergo stomatal closure in order to avoid major loss of water. Consequently, the defense barrier limits CO_2_ absorption, which ultimately hinders the light-driven processes of photosynthesis by altering enzyme such as RuBisCo and fructose-1,6-bisphosphatase (Tripathi et al., 2025). This ultimately leads to the disruption of the regeneration of the Calvin cycle and utilization of energy molecules for carbon fixation. This imbalance in the cycle further disrupts electron flow during photosynthesis, causing the leaching of electrons to oxygen which results in the formation of ROS. More specifically, singlet oxygen (^1^O_2_), which is extremely harmful to chloroplast constituents get formed when PSII is overexcited during salt stress, resulting in a decline of photosynthesis machinery (Kesawat et al., 2023). On the other hand, to prevent cellular toxicity, plants use intricate calcium-dependent signaling networks (SOS pathway) to eliminate these harmful ions (Yang and Guo, 2018). The signaling network corresponds to three phases: sensing, transduction, and execution (Ma et al., 2026). During salt sensing, Na^+^ and osmotic imbalance at the membrane occur due to membrane-associated Ca^2+^ channels and sensors (such as CNGCs and GLRs), which causes a rapid rise in cytoplasmic Ca^2+^ concentration (Demidchik and Shabala, 2018). During signal transduction, the Ca^2+^ sensor protein SOS3 (also known as CBL4) perceives the salt-induced increase in cytosolic Ca^2+^ levels and interacts with the C-terminal domain of serine/threonine protein kinase SOS2 (CIPK24). This interaction causes autoinhibition, leading to its activation. Following signal transduction, the activated SOS2–SOS3 complex phosphorylates the SOS1 transporter, which facilitates Na^+^ efflux across the plasma membrane. By maintaining cellular ion homeostasis and preventing excessive intracellular Na^+^ accumulation, this cascade plays an important role in mitigating salt-induced stress (Yang and Guo, 2018; Ali et al., 2026). Recent transcriptomic analyses repeatedly show that salt-tolerant genotypes quickly enhance expression of ion transporters, such as Na^+^/H^+^ antiporters (NHX1), present on the tonoplast, which accumulate harmful salts inside the vacuole, thereby helping in retaining water and keeping other parts of the cellular compartments protected (Ren Y. et al., 2025).

### Oxidative damage and ROS signaling pathways

Along with ion imbalance, salinity stress induces oxidative damage caused by excess accumulation of ROS. All major ROS, including hydrogen peroxide (H_2_O_2_), superoxide radicals (O_2_^∙–^), singlet oxygen (^1^O_2_), and hydroxyl radicals (OH∙) have been reported under high salt stress. These molecules are synthesized in chloroplast, mitochondria, ER, and peroxisomes during stressful conditions (Akyol et al., 2020; Amiri, 2026). During photosynthesis, two different ROS-forming processes take place in the chloroplast. The Mehler reaction occurs at Photosystem I (PSI) when electrons from the reducing molecules of the photosynthetic electron transport chain leak to O_2_, producing superoxide radicals (O_2_^∙–^), which is further dismutates by superoxide dismutase to H_2_O_2_ (Akyol et al., 2020; Amiri, 2026). The increased stimulation of chlorophyll molecules during stress at Photosystem II (PSII) results in the synthesis of triplet-state chlorophyll (^3^Chl*). This molecule transfers excitation energy to ground-state oxygen, generating highly reactive singlet oxygen (^1^O_2_) (Akyol et al., 2020). In mitochondria, superoxide (O_2_^∙–^) and H_2_O_2_ are produced through electron leakage from respiratory complexes I and III of the electron transport chain (ETC) (Liu R. et al., 2026). In the endoplasmic reticulum, oxidative protein folding also contributes to H_2_O_2_ accumulation through the formation of disulfide bonds (Liu R. et al., 2026).

In optimum condition, plants have ROS scavenging systems such as SOD, peroxidase, catalase, monodehydroascorbate reductase (MDHAR), glutathione reductase, and other compounds like phenolics, carotenoids, and tocopherols. But this well efficient scavenging system fails when the ROS accumulation is much higher than plants detoxification capacity (Akyol et al., 2020). This elevated ROS accumulation causes the peroxidation of lipids which increases the permeability of the membrane causing loss of essential minerals and ions. Normally, presence of malondialdehyde (MDA) is an indicator of lipid peroxidation and oxidative stress. In addition to this, ROS molecules also inactivate enzymes and proteins along with nucleic acids causing mutation and disruption in metabolic pathways (Boukhari et al., 2025). At regulated levels, ROS serve as crucial signaling molecules regardless their harmful nature. Respiratory burst oxidase homologs (RBOHs) often referred as NADPH oxidases, control one of the significant ROS signaling pathway by inducing ROS as a signaling molecule (Sun et al., 2025). These reactive species induce calcium influx inside the cytoplasm which in turn activate NADPH oxidases, which results in additional ROS generation. This ROS-Ca^2+^ loop act as a signaling molecule and send salinity stress signals throughout the plant’s cells (Demidchik and Shabala, 2018; Figure 3). As mentioned earlier, plants contain both enzymatic and non- enzymatic antioxidants to combat this ROS species while halophytes contain more stringent antioxidant systems which helps them to survive it under high saline environment with minimal oxidative damage (Hidri et al., 2022; Figures 1, 2). However, along with biochemical and physiological pathways, for stress mitigation plants also interact directly with rhizospheric microorganisms (Fu et al., 2025). Recent studies have shown that via helping in balancing ions, antioxidant barrier and induced stress responsive signals, many rhizospheric microorganisms carry the potential to significantly enhance plant adaptability in saline conditions. Therefore, it has become very crucial to comprehend the current understanding of rhizospheric microbiome and plant interaction under salt stress to devise sustainable methods for enhancing salt resistance of targeted crops.

**FIGURE 3:**
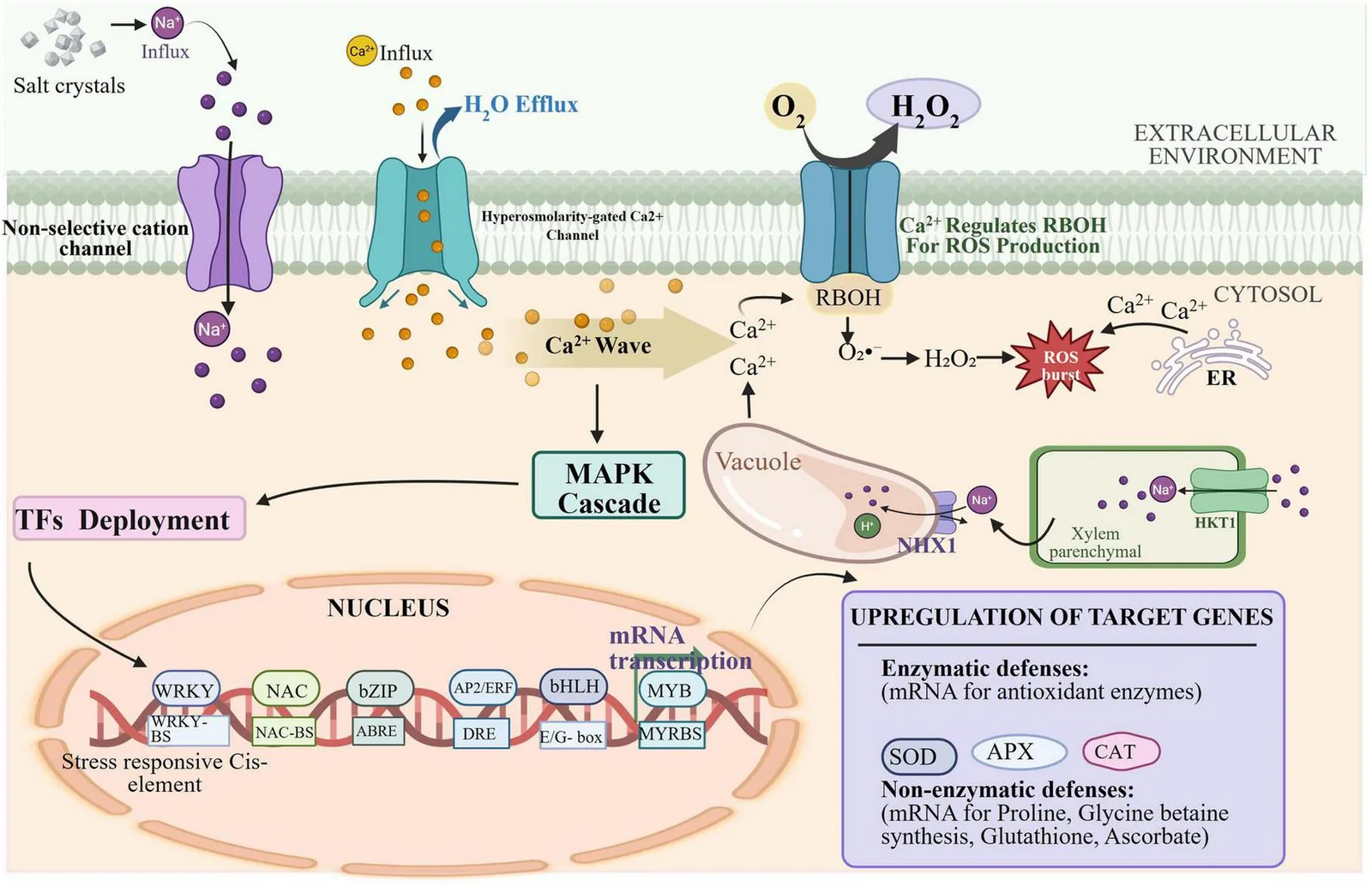
Overview of salinity stress signaling and antioxidant responses in plants. Salt stress triggers Ca^2+^ influx, ROS production, MAPK signaling, and activation of stress-responsive transcription factors (WRKY, NAC, bZIP, AP2/ERF, bHLH, and MYB), leading to enhanced antioxidant defense and osmotic adjustment. Figure was created using BioRender.

## Microbial-induced modulation of plant antioxidant systems

Salinity stress induces oxidative imbalance through excessive accumulation of ROS, which disrupt cellular integrity and metabolic function (Hasanuzzaman et al., 2021; Zandi and Schnug, 2022; Figure 3). Beneficial plant-associated microorganisms, including endophytes and, plant growth–promoting microbes (PGPM) play a critical role in mitigating salt stress by enhancing plant antioxidant capacity and stabilizing cellular redox homeostasis. These effects are mediated through coordinated regulation of stress-responsive pathways, resulting in reduced oxidative damage and improved plant tolerance under saline conditions (Radhakrishnan and Krishnasamy, 2024; Ren Y. et al., 2025).

## Activation of enzymatic and non-enzymatic antioxidants

Oxidative stress caused by salt is an important means of plant damage during exposure to high salinity (AbdElgawad et al., 2016; Balasubramaniam et al., 2023; Mishra A. et al., 2025; Mishra M. et al., 2025). This stress is associated with ionic, osmotic, and metabolic disturbances that cause the formation of reactive oxygen species (ROS) in different cell organelles such as chloroplasts, mitochondria, peroxisomes, and apoplast (Rao et al., 2025). Elevated ROS causes oxidation of lipids, proteins, and DNA, hence interfering with the normal functioning and vigor of cells (Zandi and Schnug, 2022). Plants have developed an advanced antioxidant system that includes enzymatic and non-enzymatic antioxidants. Enzymatic antioxidant defense systems of plants consist mainly of SOD, CAT, POD, and GR. At the center of this antioxidant system is the ascorbate-glutathione cycle, consisting of APX, GR, dehydroascorbate reductase (DHAR), and MDHAR ensuring redox balance within the cell (Hasanuzzaman et al., 2021; Rao et al., 2025). Furthermore, non-enzymatic antioxidants such as ascorbate, glutathione, tocopherol, carotenoid, and phenolic compounds serve as essential redox buffers. These compounds act as ROS scavengers while stabilizing cellular structure through their involvement in redox reactions. They are also involved in redox signaling pathways that regulate the oxidative-stress induced defense mechanisms. The inoculation with beneficial microbial symbionts can provide enhanced protection to the existing antioxidant system. The use of PGPM can improves the activity of the major ROS-scavenging enzymes (SOD, CAT, APX, and POD) and boosts the synthesis of non-enzymatic antioxidants (ascorbate, flavonoids, phenolics). As a result, enhanced scavenging of ROS increases the efficiency of ROS elimination along with reduced oxidative stress marker concentrations like H_2_O_2_ and MDA (Radhakrishnan and Krishnasamy, 2024; Ren Y. et al., 2025).

## Enzymatic antioxidants: mechanistic architecture and microbial modulation

Enzymatic antioxidants constitute the primary defense system that maintains cellular redox homeostasis under salinity stress (Rady et al., 2025). Their coordinated activity enables efficient detoxification of ROS and protects cellular components from oxidative damage. Beneficial microorganisms further modulate these antioxidant pathways, enhancing their efficiency and contributing to improved stress tolerance.

### Primary ROS-scavenging enzymes

First enzymatic response to ROS formation under salinity conditions is carried out by a trio of enzymes acting in substrate-linked cascade of superoxide scavenging reactions. SOD constitutes the first enzymatic barrier against salinity-induce oxidative stress by catalyzing the dismutation of superoxide radicals (O_2_^∙–^) into H_2_O_2_ and molecular oxygen (2O_2_^∙–^ + 2H^+^ → O_2_ + H_2_O_2_). Particularly, the reaction prevents formation of OH∙ due to prevention of interaction between H_2_O_2_ and O_2_^∙–^ by Haber-Weiss reaction (Rady et al., 2025). SOD enzyme can be subdivided depending on its metal cofactor into several forms: cytoplasmic and chloroplastic Cu/Zn-SOD, mitochondrial Mn-SOD and chloroplastic Fe-SOD; the distribution in the particular compartments of SOD is essential for whole cell ROS regulation under salinity. The H_2_O_2_ produced during the action of SOD should then be removed with help of CAT (EC 1.11.1.6) and APX (EC 1.11.1.11). Apart from SOD, APX, CAT, DHAR, MDHAR, GPX, glutathione S-transferase (GST) enzymes become active during saline condition as a part of detoxification signaling pathway (Zandi and Schnug, 2022). In particular, CAT enzyme is mainly peroxisomal and scarce in chloroplasts, and it directly dismutates H_2_O_2_ into H_2_O and O_2_ without reduction, thus provides high-capacity of H_2_O_2_ removal during oxidative bursts during photorespiration. The APX, by contrast, is the primary H_2_O_2_ scavenger within the chloroplast stroma and thylakoid membrane, where CAT is absent. APX possesses a strong affinity for H_2_O_2_ and is central to the ascorbate-glutathione (AsA-GSH) cycle, where it decomposes H_2_O_2_ into water using ascorbate as a reducing factor, converting ascorbate to MDHA, which is then further processed non-enzymatically to dehydroascorbate (DHA) (Cannea and Padiglia, 2025).

Microbial inoculation has been shown to modulate oxidative stress responses by reducing hydrogen peroxide accumulation while promoting the coordinated activity of key antioxidant enzymes such as SOD and CAT (Bichara et al., 2025). Consistent with this, enhanced activities of SOD, APX, and CAT, along with transcriptional upregulation of ROS-scavenging genes including CAT, APX, GR, and DHAR, have been reported in PGPR-treated *Abelmoschus esculentus* under saline conditions relative to non-inoculated controls (Habib et al., 2016). Similarly, inoculation with salt-tolerant *Enterobacter cloacae* in maize has been associated with increased expression of stress-responsive genes such as SOD and APX (Ali et al., 2022). It should be noted that microbial inoculation is not always an effective means for increasing antioxidant enzymes levels since SOD decreased after the inoculation of PGPR on maize. Thus, under certain conditions, microbial inoculation can decrease oxidative stress at the upper level so that the increased level of SOD becomes unnecessary (Ali et al., 2023). The inoculation with *Glutamicibacter* sp. and *Pseudomonas* sp., for example, is known to reduce MDA level and increase activities of antioxidant enzymes, as well as improving ionic balance through the increased level of K^+^ and Ca^2+^ and decreased Na^+^ level and stimulation of the rhizospheric enzymatic activity and phytohormones level (Hidri et al., 2022). The same result was achieved by the inoculation of *Azotobacter chroococcum* and *Alcaligenes faecalis* under salinity conditions: SOD, APX, and peroxidase activities were increased; MDA and H_2_O_2_ level were reduced; the amount of photosynthetic pigments, soluble sugars and proteins was increased, demonstrating the stabilization of metabolism (Abdel Latef et al., 2021). Additionally, microbial inoculation led to better nutrient acquisition because the level of sodium was reduced while the levels of nitrogen, potassium, calcium, and magnesium were increased (Abdel Latef et al., 2021).

### Ascorbate–glutathione (AsA–GSH) cycle as integrated redox hub in salinity stress

Despite the fact that the AsA-GSH cycle contains non-enzymatic elements as well, it is considered as an enzymatic antioxidant system because its functions rely upon the interplay between different kinds of redox enzymes responsible for H_2_O_2_ removal and antioxidants regeneration. The AsA-GSH cycle can be considered as one of the major enzymatic systems responsible for H_2_O_2_ removal in plants. It is of special significance in chloroplasts when the catalase content is low and is unable to cope with high ROS removal needs. There are four main enzymes contributing to the cycle. Firstly, ascorbate peroxidase (APX) uses ascorbate as a substrate for the reduction of hydrogen peroxide into water, thus forming monodehydroascorbate (MDHA). The latter is subsequently reduced to ascorbate via MDHAR with the participation of NAD(P)H. Alternatively, MDHA can disproportionately resulting in ascorbate and dehydroascorbate (DHA). The latter compound is reduced by dehydroascorbate reductase (DHAR) in the presence of reduced glutathione (GSH). Reduced glutathione is obtained from oxidized glutathione by the activity of glutathione reductase (GR) with NADPH involvement. This series connects H_2_O_2_ detoxification with cellular reducing power and maintains the redox state of ascorbate and glutathione pools (Tan et al., 2022; Zhu et al., 2022).

Microbial-mediated enhancement of the AsA–GSH cycle under salinity stress has been explored in limited extent, yet there are few studies demonstrating coordinated regulation of antioxidant enzymes and redox metabolites. For instance, co-application of *Bacillus subtilis* and silicon (BsSi) optimized the AsA–GSH cycle alongside increased activities of CAT and GPX, resulting in enhanced scavenging of H_2_O_2_ and superoxide radicals and reduced lipid peroxidation, as reflected by lower MDA levels under salt stress (Yu et al., 2026). Similarly, Effective Microorganisms (EM) treatment in salt-stressed bean plants increased enzymatic antioxidant activities as well as AsA and GSH contents, indicating activation of the AsA–GSH cycle as a key mechanism for detoxifying H_2_O_2_ and limiting oxidative damage (Talaat, 2014). In chickpea, subjected to salt stress, arbuscular mycorrhiza (AM), particularly in combination with silicon, enhanced both enzymatic and non-enzymatic antioxidant components and shifted the cellular redox balance toward reduced forms (GSH and AsA over GSSG and DHA), thereby strengthening redox homeostasis through efficient operation of the Halliwell–Asada cycle (Garg and Bhandari, 2016).

### The peroxidase network in salinity stress

Beyond the primary antioxidant triad and the AsA–GSH cycle, an expanded peroxidase network provides complementary and spatially distinct ROS buffering, particularly across the plant cell wall, vacuole, and apoplast, where APX and CAT are less prominent. GPX (Glutathione peroxidase; EC 1.11.1.9) play a key role in ROS detoxification by catalyzing the reduction of H_2_O_2_ and organic hydroperoxides. Unlike animal GPXs, which contain selenocysteine at their active site, plant GPXs are cysteine-based thiol peroxidases that preferentially utilize thioredoxin (Trx) rather than GSH as the reducing substrate, thereby functionally linking peroxide detoxification to the thioredoxin antioxidant system (Bela et al., 2022). Mechanistically, GPXs operate through oxidation of a catalytic cysteine residue to sulfenic acid, followed by disulfide bond formation and subsequent reduction by Trx, enabling both peroxide detoxification and participation in redox signaling (Wang et al., 2024). Consistent with this dual functionality, plant thiol peroxidases contribute to both ROS scavenging and signal transduction processes (Bela et al., 2022).

In contrast, Class III PODs (peroxidases) constitute a large multigene family predominantly localized in the apoplast and cell wall, where they oxidize phenolic substrates in H_2_O_2_-dependent reactions, thereby coupling ROS detoxification with cell wall lignification and structural reinforcement under stress conditions (Veljović Jovanović et al., 2018). Guaiacol peroxidase (GPOX; EC 1.11.1.7) activity, commonly used as a proxy for this class is often co-induced with SOD and CAT in microbially inoculated plants. Under saline conditions, inoculation with the rhizobacterial strain *Pseudomonas taiwanensis* (PK7; Accession no. MT218377) significantly enhanced antioxidant enzyme activities in rice seedlings, with GPOX showing the highest induction (46.5-fold), followed by CAT (7.5-fold), SOD (4.7-fold), and APX (3-fold) (Reshma and Dileep, 2025). The greater increase in GPOX activity here indicate that Class III peroxidases also play prominent role in microbially mediated oxidative stress mitigation under salinity stress. As various antioxidant enzymes get involved, their role and prominence might depend on factors like plant and microbial system involved, level and quality of salt stress and likewise.

## Non-enzymatic antioxidants: integrated redox buffers and metabolic defenses under salinity stress

Non-enzymatic antioxidants constitute a highly dynamic and multifunctional arm of the plant defense system, operating in parallel with enzymatic machinery to maintain cellular redox homeostasis under salinity stress. Unlike enzymatic antioxidants, which catalyze discrete detoxification reactions, non-enzymatic components function as redox buffers, electron donors, osmoprotectants, and signaling mediators, enabling both direct scavenging of ROS and modulation of redox-sensitive pathways. Salinity-induced oxidative stress driven by ionic imbalance and osmotic perturbations leads to excessive accumulation of ROS such as superoxide radicals, hydrogen peroxide, and hydroxyl radicals, necessitating a robust non-enzymatic buffering system to prevent oxidative damage to lipids, proteins, and nucleic acids (Hasanuzzaman et al., 2021; Zandi and Schnug, 2022). These metabolites are compartmentally distributed across chloroplasts, mitochondria, cytosol, vacuole, and apoplast, ensuring spatially coordinated protection and signaling under stress conditions.

### Core redox buffers: ascorbate and glutathione

The centerpiece of this mechanism is ascorbate (AsA) and glutathione (GSH), which contribute to the cellular redox environment. AsA is a potent, water-soluble antioxidant that directly neutralizes ROS and performs photoprotection, lipid-phase antioxidants regeneration, and signaling redox pathway regulation. The redox status of AsA, assessed by the AsA/DHA ratio reflects the balance between reduced AsA and its oxidized form DHA and indicates the cellular oxidative stress and is tightly correlated with stress tolerance in plants. In plants, limited amounts activate stress-responsive signaling pathways and help the organism adapt to the negative environmental factors; however, excessive amounts have been reported to cause damage to cellular structure and metabolism (Xiao et al., 2021). GSH is a thiol-based redox buffer that maintains the intracellular redox potential and protects protein thiols from irreversible oxidation. Similarly to AsA, the levels of glutathione also get correlated in terms of the sizes of its pools and their degree of oxidation, rather absolute concentration, that will have differentiated response under degree of stress conditions. The GSH/GSSG ratio is an important indicator of cellular redox balance, similar to AsA/DHA and affects detoxification, protein stability, and glutathionylation reversible signaling (Aoyama and Nakaki, 2015). During salinity stress, maintenance of a highly reduced GSH pool is strongly associated with increased metabolic stability and stress tolerance (Garg and Bhandari, 2016; Siddika et al., 2024). Thus, the AsA/DHA and GSH/GSSG couples represent distinct but metabolically interconnected components of cellular redox regulation (Foyer and Kunert, 2024). Beneficial microorganisms support the AsA-GSH cycle by maintaining the high concentrations of reduced AsA and GSH, increasing the activity of APX, GR, DHAR, and MDHAR, as well as maintaining the beneficial AsA/DHA and GSH/GSSG ratios during salinity stress. In such a way, microbial inoculation increases H_2_O_2_ detoxification, preserves the redox balance of the cell, and reduces the harmful effect of excessive ROS production. Therefore, interactions between beneficial microorganisms under salinity stress affect the antioxidant metabolism and efficiency of the AsA-GSH cycle (Talaat, 2014; Garg and Bhandari, 2016; Yu et al., 2026).

### Osmoprotectants with antioxidant functions

In addition to classical redox buffers, several osmoprotectants contribute significantly to oxidative stress mitigation under salinity by functioning as compatible solutes with antioxidant properties. Proline is one of the most extensively studied osmolytes, accumulating to high levels under salt stress. Beyond osmotic adjustment, proline acts as a ROS scavenger, stabilizer of proteins and membranes, and regulator of redox balance, while its metabolism is closely linked to mitochondrial redox status and NADP^+^/NADPH cycling. Proline accumulation has been associated with reduced lipid peroxidation and enhanced stress tolerance (Mushtaq et al., 2025). Glycine betaine (GB) is another key osmoprotectant that stabilizes macromolecular structures, particularly proteins and membranes, under osmotic stress. GB protects the photosynthetic apparatus by maintaining the integrity of photosystem II and reducing ROS generation, while also indirectly enhancing antioxidant enzyme activities and redox homeostasis (Basit et al., 2025). Trehalose, a non-reducing disaccharide, functions as both an osmoprotectant and signaling molecule, protecting cellular structures through stabilization of membranes and proteins while also modulating stress-responsive gene expression. Trehalose metabolism is closely linked to energy signaling pathways and has been shown to enhance antioxidant capacity and ROS scavenging under salinity stress (Yang et al., 2022).

Microbial inoculation can also modulate osmolyte accumulation in salt-stressed plants. PGPR and mycorrhizal associations have been reported to alter the accumulation of osmolytes such as proline, glycine betaine, and trehalose in salt stressed plants. Thus, potentially contributing and supporting cellular hydration, protecting macromolecular structures, and sustaining water uptake. For example, hydroponic lettuce exposed to 150 mM NaCl showed an approximately ninefold increase in proline relative to unstressed plants, and this response was moderated by biostimulant treatment (Zuzunaga-Rosas et al., 2024). Similarly, under 50 mM NaCl, PGPR inoculation increased plant leaf proline levels by 22% while reducing lipid peroxidation by 41%–42% (MDA) relative to uninoculated salt-stressed plants (İkiz et al., 2024). Arbuscular mycorrhizal fungi (AMF) inoculation has also been associated with increased glycine betaine accumulation in plants under salinity, with AMF-associated *Phragmites australis* plants showing up to a 2-fold increase at higher NaCl levels (Evelin et al., 2009). Because of differences in host, salinity regime, inoculant, exposure duration, tissue, and experimental system the responses and their degree vary. The values are therefore reported as study-specific observations, not comparable effect sizes. Importantly, increased osmolyte concentrations in plant tissues following microbial inoculation cannot be used to demonstrate microbial synthesis or transfer to the host. Under stress, plant associated microbes themselves needed to protect themselves and for that different strategies get applied, including formation of many protective compounds. Some endophytic and halotolerant bacteria can synthesize compatible solutes such as ectoine, trehalose, proline, glycine betaine, and Nε-acetyl-β-lysine for their own osmotic adaptation. Similarly, many species including *Bacillus cereus*, *Micrococcus yunnanensis*, and *Enterobacter tabaci* have been shown to enhance antioxidant defense and osmolyte production and improve salt tolerance in crops such as rice, soybean, maize, and tomato (Kamran et al., 2022), and seed coating with endophytic bacteria further supports osmotic balance and reduces oxidative stress under saline conditions (Gong et al., 2024). Yet, the effect of microbially produced molecules on host plant and resulting probable transfer to the plant is still work in the progress. For example, *Halolamina pelagica* CDK2 produces trehalose and improves wheat performance under saline stress, but the amount of trehalose transferred from the microbe to the host tissue was not measured (Naitam et al., 2023). Emerging techniques like isotope-labeling and osmolyte-synthesis mutants of the inoculant strain would be helpful in near future (Hui et al., 2025).

### Lipophilic antioxidants: membrane protection and photoprotection

Lipophilic antioxidants play a critical role in protecting cellular membranes and photosynthetic structures from oxidative damage. Microbial inoculation helps plants better cope with salinity stress by strengthening their lipid-based antioxidant defense system. In inoculated plants, higher levels of α-tocopherol (Vitamin E) protect cell membranes by scavenging lipid radicals and reducing membrane damage (Kukri et al., 2025). At the same time, increased carotenoid content supports both light harvesting and photoprotection by quenching reactive oxygen species like singlet oxygen. Microbial inoculation with plant growth–promoting bacteria (PGPB; *A. chroococcum*, *Enterobacter asburiae*, and *Lactococcus lactis*) and the endophytic fungus *Piriformospora indica* enhances the accumulation of lipophilic antioxidants under salinity stress. When inoculated, wheat plants show increased α-tocopherol levels and carotenoid content and were associated with reduced MDA content and LOX activity, indicating improved membrane integrity and lowered oxidative damage (Singh and Tiwari, 2021). Similarly, microbial consortia comprising *Pseudomonas koreensis*, *Pseudomonas vancouverensis*, *Pseudomonas putida*, and *Pantoea agglomerans* in floating hydroponic lettuce significantly increased carotenoid levels (2–4-fold), along with enhanced chlorophyll content and biomass, further supporting microbe-mediated enhancement of non-enzymatic antioxidant systems (Molaei et al., 2024). Thus, microbes can function as critical modulators of lipophilic antioxidant networks, reinforcing membrane stability and photoprotection to sustain plant performance under salinity stress.

## Reduction of ROS and protection of cellular structures

The antioxidant systems described in above section do not operate in isolation, their ultimate purpose is to shield the physical structures of the cell from oxidative destruction. Salinity stress attacks membranes, chloroplasts, mitochondria, proteins and DNA simultaneously. If these structures are not physically protected, even a well-activated biochemical defense system cannot sustain plant survival. Therefore, how beneficial microbes arm the plant’s ROS-scavenging machinery and what cellular structures are protected as a result under salt stress is needed to understand.

## Enzymatic antioxidant defense: arming the first line

Microbial inoculation has the potential to modulate the plant enzymatic antioxidant system that represents a coordinated network of enzymes that detoxify ROS. The direction and extent of enzymatic antioxidant responses are affected by the particular strain of microbe, the host species, the level of salinity, the tissue sampled, and the time of sampling. For instance, in tomato plants under high NaCl, *Sphingomonas* sp., *Pseudomonas pseudoalcaligenes*, and *Bacillus pumilus* significantly elevated SOD, CAT, APX, GR, and GST activities, accompanied by lower markers of lipids, proteins, and nucleic acids from ROS-induced oxidative damage (Kumar et al., 2020). Cyanobacteria: *Plectonema boryanum*, and *Oscillatoria acuta* regulated ROS in rice leaves by modulating production of peroxidase enzymes in host plants (Yadav et al., 2022). EM applied to common bean (*Phaseolus vulgaris*) at up to 5.0 dS m^–1^ salinity improved the ROS-scavenging enzyme system, modified protein and polyamine pools, and maintained cellular integrity throughout the growing period (Talaat, 2019).

At the molecular regulatory level the microbes carry the potential to influence the plant’s genetic circuitry. The *Pseudomonas fluorescens* enhances the expression of miR398 in tomato plants, a microRNA that upregulates *CuZn superoxide dismutase 1* (*CSD1*) and related antioxidant genes, facilitating sustained ROS detoxification and redox homeostasis (Salunke et al., 2025). In rice, *B. subtilis* modulates miR399 and lncRNA1459 targeting ion transporter genes HKT1 and NHX1, reducing Na^+^ toxicity and simultaneously enhancing antioxidant enzyme activity at the cellular level, while in *Arabidopsis*, miR398b and miR398c were found being heavily downregulated under fungal endophytes with inverse relationship with gene expression of antioxidant enzymes (Salunke et al., 2025; Mishra A. et al., 2025). Functional perturbation experiments, such as those involving genetic mutants provide further evidence on role of hormone biosynthesis and signaling genes involved under microbial influence. For example, volatiles from *Bacillus amyloliquefaciens* FZB42 increased shoot fresh weight and lateral root number in *Arabidopsis* under 100 mM NaCl, but these effects were not significant in the jasmonate-related mutants jar1-1 and myc2 (Liu et al., 2020). In *Bradyrhizobium japonicum* IRAT FA3, the promotion of the shoot biomass under salt stress was only observed in wild-type Arabidopsis and not in jar1 and myc2 mutants (Gomez et al., 2023). In tomato, the growth-promoting effect of *B. subtilis* CNBG-PGPR-1 under salinity was reduced by inhibition of ethylene biosynthesis with aminoethoxyvinylglycine (AVG), and the observed microbial-induced tolerance was abolished by inhibition of ethylene signaling with AgNO_3_ (Feng et al., 2024). Therefore, functional evidence indicates that host jasmonate and ethylene signaling along with miRNAs get modulated under microbial-association under salinity with role in influencing and regulating antioxidant machinery for plant adaptation.

## Non-enzymatic antioxidants: the secondary shield

Alongside enzymatic defense, beneficial microbes also strengthen the plant’s non-enzymatic antioxidant system by enhancing the accumulation of metabolites such as ascorbate, GSH, tocopherols, carotenoids, phenolics and flavonoids. These compounds collectively reinforce cellular redox homeostasis, protect membranes from oxidative damage, and complement enzymatic ROS detoxification under salinity stress (Karimi et al., 2022). In quinoa (*Chenopodium quinoa*) under salinity, combined application of *Trichoderma harzianum* and *Azotobacter vinelandii* significantly elevated total phenol and flavonoid content above control treatments, with the highest phenol levels recorded at 300 mM NaCl under *Trichoderma* treatment (Karimi et al., 2022). The application of *Halothiobacillus halophilus* to *Plantago coronopus* enhanced both glutathione and phenolic compounds, increasing antioxidant defense and salt tolerance (Salunke et al., 2025). The *Bacillus* spp. inoculation in *Solanum lycopersicum* increased not only antioxidant enzyme activity but also chlorophyll and carotene content, directly reducing cell membrane damage via ROS reduction (Yoo et al., 2019).

## Plasma membrane integrity: the first barrier of physical defense

The plasma membrane is the first structure to bear the consequences of salinity stress. ROS-driven oxidation of membrane phospholipids (lipid peroxidation) disrupts membrane organization, increases ion leakage and ultimately disables the transport proteins that regulate Na^+^ and K^+^ balance. Membrane damage is often evaluated by measuring the accumulation of malondialdehyde (MDA) as a biochemical marker for lipid peroxidation, and electrolyte leakage as an indirect indicator of membrane permeability and cell damage (Chakraborty et al., 2024; Morales and Munné-Bosch, 2024). PGPR application in salt-stressed plants has been shown to significantly reduce MDA content and electrolyte leakage while simultaneously restoring K^+^/Na^+^ balance (Sharma et al., 2021). The responses suggest diminished lipid peroxidation via these indirect measurements. A systematic review of 174 studies confirmed that PGPR treatment reliably decreased MDA and electrolyte leakage across a wide range of crop species under both drought and salinity conditions (Kibret et al., 2024). As Na^+^ imbalance can lead to shrinkage of associated membrane and therefore associated physical deformation, EPS mediated Na^+^ adsorption also considered to helping the cell adaptation. Halotolerant bacteria have been implicated to create hydrated microenvironment that may act as buffer against osmotic shock at the cell surface (Liu et al., 2022). The EPS produced by *Pseudomonas simiae* MHR6 showed Na^+^ adsorption activity and its application resulted in improved Na^+^/K^+^ homeostasis in maize under salt stress (Liu et al., 2022). As EPS is major source of biofilm formation by bacteria, in *B. pumilus* FAB10, biofilm formation were suggested to help mitigate adverse effect of saline condition on wheat roots (Ansari et al., 2019). However, the proposed association between biofilm formation, rhizosphere Na^+^ immobilization, and enhanced water retention leading to preservation of plant cellular structure under salinity stress requires further experimental validation.

## Chloroplast protection and photosynthetic recovery

The chloroplast is particularly vulnerable to salinity because it is both a site of active ROS production and the organelle most dependent on precise membrane architecture to function. Salinity disrupts thylakoid membrane continuity, damages the photosystem II (PSII), and degrades chlorophyll and carotenoid content, all of which reduce the plant’s ability to capture and convert light energy. The most widely used indicators of PSII photochemical performance include the maximum photochemical efficiency of PSII (Fv/Fm), the variable-to-minimum fluorescence ratio (Fv/Fo), and the photochemical quenching coefficient (qP), all of which are significantly reduced under salinity stress (Boorboori and Lackóová, 2025). The chlorophyll content and Fv/Fm of AMF-inoculated *Diospyros lotus* seedlings were higher than those of the uninoculated ones under salinity stress (Incesu et al., 2025). When thylakoid membranes are intact and the plastoquinone redox cycle functions normally, PSII is shielded from singlet oxygen damage and photoinhibition is avoided (Vetoshkina et al., 2024; Boorboori and Lackóová, 2025). In above example, AMF achieve better chloroplast physiology through increased transcript levels of chloroplast genes encoding antenna proteins involved in excitation energy transfer, allowing the photosynthetic apparatus to repair and sustain itself even under ongoing saline pressure (Evelin et al., 2019). In *Robinia pseudoacacia* under saline conditions, AMF colonization correlated with enhanced transcript accumulation of RppsbA in response to salinity, whereas RppsbD transcript levels were up-regulated by 100 mM NaCl (Chen et al., 2017). These findings suggest that the transcription of PSII-associated genes get modulated.

Effective Microorganisms applied to common bean under saline conditions increased the maximum quantum efficiency of PSII, actual photochemical efficiency, electron transport rate, and photochemical quenching as compared to uninoculated plants under salinity stress, indicating an enhancement in the photochemical performance of PSII under salt stress conditions (Talaat, 2019). Root metabolite profiling revealed the presence of choline, a precursor of glycine betaine and phospholipids, in rice treated with *P. taiwanensis* PK7 (Reshma and Dileep, 2025). Under inoculation of soybean plants with ALT29 and ALT43 halotolerant microbial strains, upregulation of the *GmFLD19* gene was directly linked to reduced Na^+^ and MDA levels relative to the uninoculated salt-stressed plants (Khan et al., 2021). The interconnected mechanisms underlying plant–microbe interactions under salinity stress, together with their multi-omics perspectives, are summarized in Figure 4.

**FIGURE 4:**
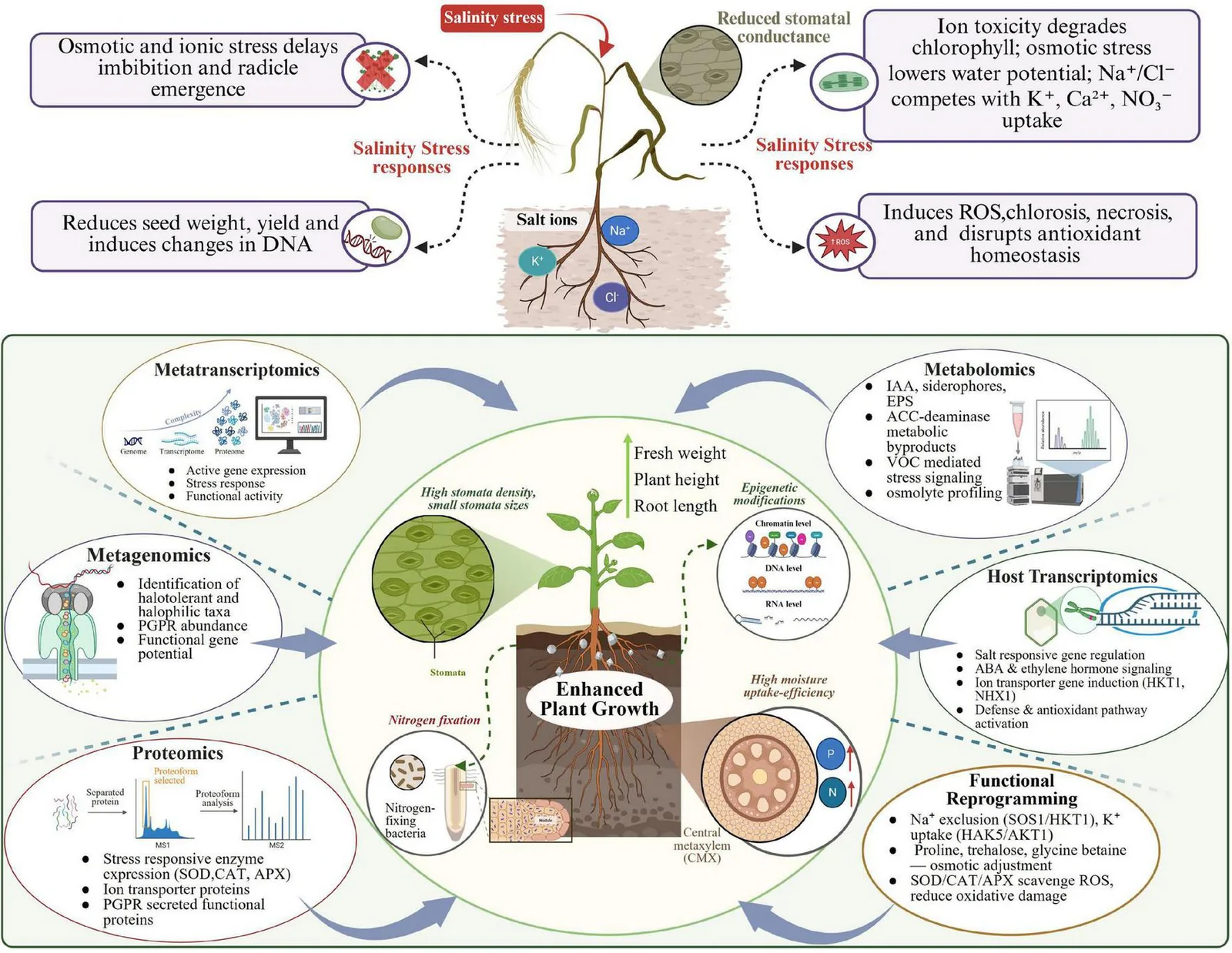
Integrated multi-omics strategy for mitigating plant salinity stress using PGPR (plant growth-promoting rhizobacteria). Salinity stress disrupts plant physiology through excess Na^+^/K^+^/Cl^–^ ion accumulation, leading to inhibited seed germination and root growth, reduced stomatal conductance, decreased chlorophyll content and nutrient uptake, ROS accumulation with oxidative damage (chlorosis, necrosis), and reduced seed yield with genotoxic DNA effects. A multi-omics framework; metagenomics, metatranscriptomics, proteomics, metabolomics, host transcriptomics, and functional reprogramming, is applied to characterize and harness halotolerant PGPR communities and their salt-tolerance mechanisms (e.g., Na^+^ exclusion via SOS1/HKT1, K^+^ uptake via HAK5/AKT1, osmolyte accumulation, ROS-scavenging enzymes, ACC-deaminase and phytohormone production, epigenetic regulation). Together, these mechanisms enhance plant salt tolerance, resulting in increased fresh weight, plant height, root length, stomatal regulation, and nutrient/water-uptake efficiency. Figure was created using BioRender.

## Omics insights into plant-microbe-salt interactions

The utilization of omics techniques has given significant insight on interactions between plants, microbes, and stress environments including salt stress. This knowledge enables to shift focus away from focusing on single gene or organism, individually, to an integrated assessment at the level of community structure, gene expression, proteins, and metabolites (Ren Y. et al., 2025). For example, genomics approaches discover candidate genes from both host and microbes while metagenomics provide the idea of microbial composition and diversity under a particular micro-ecosystem. Integration of different omics approaches aims to link these measurements through shared biological processes and evaluate their connections to ion homeostasis, oxidative stress, and growth in the host. For instance, the combined analysis of bacterial and plant transcript profiles suggested that sulfur metabolism contributes to the salt tolerance conferred by *Enterobacter* sp. SA187; and functional analyses in *Arabidopsis* sulfur-assimilation mutants validated the claim (Andrés-Barrao et al., 2021).

## Microbial diversity shifts/key microbial taxa and their functional roles in salt-stressed rhizospheres

The mechanism and deftness to counter stress acts as a selection criterion that affects the microbial community diversity within the rhizosphere. Under salinity stress the effect can be detected both at alpha and beta diversities (Yang et al., 2023). Often, there is a decrease in Shannon diversity and observed operational taxonomic units (OTUs) in the rhizosphere under NaCl stress under 16S based amplicon sequencing, while there is an increase in the overall diversity of soil due to the heterogeneity of micro-environments induced by ionization. Rice rhizospheres subjected to salt stress shows that the bacterial richness and diversity could become significantly lower with increase in changes in the relative abundance of *Chloroflexi*, *Proteobacteria*, and Actinobacteria and decreases in Firmicutes, Acidobacteriota, and Myxococcota. While, proportion of Firmicutes, Acidobacteriota, and Myxococcota get decreases with an increase in the number of Bacteroidota (Bacteroidetes) and Cyanobacteria. Due to the crucial importance of Chloroflexi, being involved in carbon turnover, it was most prevalent phyla (Zhou et al., 2024). Rhizospheric microbiome clustered using the Bray-Curtis dissimilarities and the UniFrac distances showed clear clusters in PCoA. Soil electrical conductivity emerging as a primary environmental variable explaining community compositional variance (Hou et al., 2021). The affected taxa improve plant salt tolerance (Zhou et al., 2024). Similarly, the taxa associated with salinity in maize fields are candidate indicators of saline soil conditions (Hou et al., 2021). To validate their use as biomarkers, they would need to be tested on different samples, while their contribution to plant salt tolerance would require manipulating the microorganisms and measuring the plants’ response. Field experiments conducted on saline-alkali soil indicated that soil amendments could be able to alter soil physicochemical properties and bacterial community composition, suggesting that changes in soil environment would lead to shifts in the microbial community under salt stress conditions (Zhao et al., 2025). In a related study, performed in a saline mangrove ecosystem, the assembly and succession of leaf endophytic diazotroph communities were found to be connected to the dispersal processes and nutrient availability, with total sulfur being an important environmental variable (Hou et al., 2026). The data suggest the role of the physicochemical environment and nutrient availability in shaping the microbial community within the host-associated or soil habitat in response to saline stress.

In addition to soil physicochemical properties, host genotype and root exudation also play a role in shaping the structure of microbial communities in response to salinity stress. Indeed, in perennial ryegrass, salt-sensitive and salt-tolerant genotypes exhibited divergent root exudate profiles, and several exudate groups were correlated with changes in bacterial abundances under saline conditions (Cao et al., 2024). Therefore, salinity-induced patterns in microbial communities should be considered in the context of host genotype, root exudation, soil physicochemical properties, and study-specific moisture, climatic, and management conditions. For instance, salinity altered the microbial community of *Avicennia marina* by reducing the relative abundance of Proteobacteria while increasing the abundance of Bacteroidota in the phyllosphere and Actinobacteriota in the rhizosphere, reflecting the greater adaptation of these taxa to saline environments (Yang et al., 2023). Apart from taxonomic changes, salinity can affect functional potential. For example, the increased salinity levels in the *A. marina* ecosystem led to the increased abundance of catalase, alkaline phosphatase, and 3-phytase predicted enzymes in the phyllosphere and rhizosphere (Yang et al., 2023). The same tendency was observed in the case of the rice plant, where, with the increased salinity, chemoheterotrophic and aerobic chemoheterotrophic activities was suppressed (Zhou et al., 2024). At the molecular level, salinity induces transcriptional regulation of EPS biosynthesis genes such as exoK and exoM in *Rhizobium radiobacter* SZ4S7S14, demonstrating that EPS production is a dynamic microbial adaptation to osmotic stress (Rasulov et al., 2020). At the individual taxon level, there is evidence of specific organisms playing a crucial role in promoting the health and growth of plants in the process of overcoming salinity. For example, it should be mentioned that *Kushneria konosiri* (epiphyte) living on leaves and *Bacillus marisflavi* (endophyte) living in the root zone as symbionts are vital for the growth of many plants in a saline marine environment (Yang et al., 2023).

The structure of fungal community also experiences significant changes due to increased salt concentrations in soil. The AMF from Glomeromycotan group has been shown to provide tolerance to high levels of salinity, even though, often, high salt stress is known to negatively affect AMF colonization through interference with spore germination, mycelial development, and formation of arbuscules (Boorboori and Lackóová, 2025). The colonization of *Glomus clarum* in *D. lotus* decreased 51% in the absence of NaCl and 18% at 150 mM NaCl, yet, the dry mass and phosphorus-sodium concentration in leaves were reported to either stable or enhanced under salinity (Incesu et al., 2025). As, the result did report the presence of AMF, even at reduced level, there could be possible trade-off between carbon and nutrients need of microbes combined with efficiency and more active metabolic machinery under duress that result in better plant health under stress under microbial influence. In one study, isotope tracing technique indeed found that the ratio of phosphorus acquisition to carbon allocation by the host varied with soil phosphorus status playing role (Lekberg et al., 2024). Many plant-associated fungi (such as *Trichoderma*) thrive in salty environments owing to their high competitiveness and use of carbon sources provided by plants (Macías-Rodríguez et al., 2018). At the mechanistic level, the aforementioned ecological selection exerted by AMF correlates with their abilities to affect Na^+^/K^+^ homeostasis via ion selectivity, increase nutrient acquisition by elevating absorption of potassium (K^+^), calcium (Ca^2+^), and magnesium (Mg^2+^), and enhance osmotic adjustments by increasing accumulation of organic compounds (e.g., proline and glycine betaine). The discussed effects become possible via activation of transporter systems (NHX and SOS pathways), as well as stress response cascades allowing plants to maintain physiological functions in conditions of saline stress (Boyno et al., 2025).

Archaea are worth considering in saline plant-microbe systems due to their different ecological responses to salinity and evidence of their capacity to improve plant growth in saline soils. In 63 sites in the Hetao Plain, the variations in soil salinity were related to differences in the abundance of bacteria and Archaea (Zhang et al., 2024). In 20 Tibetan alpine wetlands, the diversity of bacteria decreased with increasing salinity, while Archaea diversity decreased and then increased (Liu X. et al., 2026). These findings reflect the response of these communities to different environmental conditions; however, the reports do not show that these microbes improve the salt tolerance of plants. Experimental studies on inoculated plants provide information on the benefits of Archaea. For instance, *H. pelagica* CDK2 showed increased vegetative growth and yield of wheat in a pot experiment with non-sterile soil kept at 8 dS m^–1^ (Naitam et al., 2023). The findings indicate that the microbe improves the growth of the crop under the tested conditions; however, the mechanism and performance under actual field conditions require further research.

## Functional reprogramming of the rhizosphere microbiome under salinity

Salinity induces profound functional reprogramming of rhizosphere microbial communities, shifting their metabolic activities from growth-oriented processes toward stress adaptation and cellular homeostasis. Shotgun metagenomics has enabled direct characterization of these functional changes by revealing genes and pathways associated with ion transport, osmotic regulation, and stress tolerance in saline ecosystems (Wang et al., 2026). The difference between “growth expansion” and “homeostasis maintenance” is thus taken as a model for interpreting the shift in functional potential, rather than as a basis for inferring changes in microbial activity. In *Tamarix*, the increase in salinity was correlated with an increase in the abundance of genes involved in ion homeostasis such as Na^+^/H^+^ antiporters and V/A-type ATPases and genes involved in compatible-solute biosynthesis, such as betaine (betA/B/C) and proline (proA/B/C) (Wang et al., 2026). Metatranscriptomic or metaproteomic measurements would be required to determine whether these functions are actively expressed under the tested conditions. Host genotype also influences microbial functional potential under salinity. Salt-tolerant grapevine genotypes exhibit enrichment of sulfur metabolism, glutathione metabolism, bacterial chemotaxis, and sulfur metabolism genes (cys, sox, and sqr), indicating enhanced detoxification capacity and metabolic versatility. These functional traits are accompanied by enrichment of plant growth-promoting bacterial groups including Planctomycetes, Chloroflexi, Verrucomicrobiota, Firmicutes, and Bacteroidetes (Wang B. et al., 2023). All in all, salt-induced restructuring of the microbiome takes place due to the concerted reprogramming of functions. Environmental filtering and genotype effects work together to favor certain microbial traits that facilitate the adaptation of host organisms to salt stress conditions.

## Microbiome profiling approaches: 16S rRNA amplicon sequencing and shotgun metagenomics

The 16S rRNA gene sequencing of amplicons remains the most common tool used in microbial community profiling, which is possible due to the scalability and ability to rely on well-curated reference databases like SILVA (Zhao et al., 2023; Ramakodi, 2022). Fungal community profiles are commonly based on internal transcribed spacer (ITS) regions, although the accuracy of these spacers for classifying fungal taxa varies among taxonomic groups and is affected by the quality and extent of reference sequence databases (Schoch et al., 2012). However, the results of the analysis depend largely on certain aspects of the chosen methodology, including, among others, the choice of a database or a particular combination of primers. It can be noted that different databases affect significantly not only taxonomic predictions but also alpha and beta diversity estimations and identification of core microbiomes (Ramakodi, 2022). In addition to database selection, primer choice also introduces methodological bias. The choice of primers can affect not only the predicted richness of a microbial community but also the relative abundance of specific bacterial and archaeal groups, thereby making comparison of community profiles generated with different sets of primers challenging (Zhao et al., 2023).

In contrast, shotgun metagenomic sequencing avoids target-specific amplification of a selected 16S rRNA region, reducing the influence of primer selection and allowing broader characterization of microbial genes and their potential functions. These annotations describe the genetic potential represented in community DNA; they do not measure transcription or metabolite production. The comparison of shotgun metagenomic and amplicon data demonstrates that despite some similarities in the analysis of community diversity across an environmental gradient, there exist considerable differences between them concerning abundance estimates and resolution levels (Zhao et al., 2023). However, the shotgun metagenomic data can be subject to biases introduced during the DNA extraction and other parts of the analysis workflow, which have the potential to influence the inferred abundance of the different members within microbial communities (McLaren et al., 2019). Thus, it would be best to consider these methods as complementary, where the analysis strategy is driven by the question being asked instead of claiming one approach is objectively more accurate or less biased than the other. Amplicon sequencing is generally less expensive and more scalable for broad community surveys, whereas shotgun metagenomics provides gene-level functions and finer taxonomic resolution if sufficient sequencing depth is available, at greater sequencing and computational cost. Neither method directly measures microbial activity, and so the choice of a method needs to be guided by the biological objective (Table 1).

**TABLE 1:** Omics and multi-omics studies elucidating plant-microbe interactions under salinity stress.

Plant system	Microbial partner/community	Omics approach	Major molecular findings	Salinity tolerance outcome	References
Maize *(Zea mays)* rhizosphere and bulk soil	Native soil bacterial and fungal communities	16S rRNA and ITS amplicon sequencing	Salinity-induced microbial community restructuring characterized by enrichment of Firmicutes, Bacteroidetes, and Deinococcus–Thermus, with decline of Acidobacteria, Chloroflexi, Nitrospirae, and Rokubacteria. Community composition was strongly associated with Ca^2+^, electrical conductivity (ECe), available phosphorus (AP), and available potassium (AK). Firmicutes and Bacteroidetes emerged as key salinity-responsive microbial indicators.	Selection of salt-adapted microbial communities and enrichment of taxa with potential plant growth-promoting and stress-resilience traits under saline conditions.	Hou et al., 2021
Salt-tolerant grapevine genotypes	Rhizosphere bacterial communities	Comparative shotgun metagenomics	Enrichment of PGPR-associated taxa (Planctomycetes, Bacteroidetes, Verrucomicrobiota, Cyanobacteria, Firmicutes), sulfur and glutathione metabolism pathways, bacterial chemotaxis, and sulfur-cycle genes (*cysNC, cysQ, sat, sir, fsr, soxB, sqr*).	Enhanced oxidative stress mitigation, microbial recruitment, sulfur cycling, and rhizosphere-mediated salt adaptation.	Wang B. et al., 2023
*Avicennia marina* (mangrove)	Rhizosphere and phyllosphere microbiomes	16S rRNA and ITS amplicon sequencing with functional prediction (PICRUSt2)	Salinity-driven shifts in microbial community structure; decreased Proteobacteria and increased Bacteroidota and Actinobacteriota under high salinity. Enrichment of stress-responsive and nutrient-cycling functions including catalase, alkaline phosphatase, acid phosphatase, and 3-phytase. Isolation of salt-tolerant PGPR (*Kushneria* and *Bacillus*) with growth-promoting activity.	Recruitment of salt-adapted microbiota, enhanced oxidative stress mitigation, improved phosphorus utilization, and increased plant fitness under saline conditions.	Yang et al., 2023
*Oryza sativa* (rice)	Rhizosphere bacterial communities	16S rRNA amplicon sequencing and functional prediction (FAPROTAX)	Salinity reduced microbial diversity and reshaped rhizosphere assembly, enriching salt-tolerant taxa (Bacteroidota, Cyanobacteria, Azospirillaceae, Tropicimonas) while suppressing Firmicutes, Acidobacteriota and Myxococcota. Functional shifts were observed in chemoheterotrophic, phototrophic and nitrogen-cycling processes, indicating microbiome adaptation to saline environments.	Recruitment of salt-adapted microbial communities with enhanced ecological resilience under saline conditions.	Zhou et al., 2024
Licorice (*Glycyrrhiza uralensis*)	*Bacillus subtilis*	Integrated transcriptomics and metabolomics	Coordinated regulation of arginine and proline metabolism, phenylpropanoid biosynthesis, glutathione-mediated antioxidant defense, and cell wall modification pathways. Multi-omics integration identified differential expression of *XET, GPX, COMT, PAL, CCR*, and POD-related genes, coupled with accumulation of osmoprotective and antioxidant metabolites.	Enhanced osmotic adjustment, ROS detoxification, cell wall stability, and salinity tolerance.	Xiao et al., 2024
Rice (*Oryza sativa*) paddy soil methanogenic microbiome	Methanogenic archaea (*Methanosarcinaceae, Methanocellaceae, Methanomassiliicoccales*) and fermentative bacteria (*Clostridiaceae*)	Double-RNA metatranscriptomics (16S rRNA + mRNA)	Salinity-induced enrichment of Clostridiaceae and suppression of Methanocellaceae activity; downregulation of CAZyme-mediated cellulose/xylan degradation and hydrogenotrophic methanogenesis, coupled with enhanced glycolysis, pyruvate-to-acetate conversion, ethanol fermentation, and methylotrophic methanogenesis (*mtaAB*). Increased expression of osmoadaptation pathways including choline uptake–betaine synthesis, Nε-acetyl-β-lysine biosynthesis (ablB), flagellin biosynthesis, and V/A-type H^+^/Na^+^ ATPase-associated functions.	Enhanced microbial osmoprotection, maintenance of acetoclastic methanogenesis, and metabolic resilience of the anaerobic food web under salinity stress.	Zhou et al., 2025
Mangrove (*Avicennia germinans*)	Endophytic microbiome of leaves and pneumatophores (root-associated bacteria, fungi, archaea)	Integrated metagenomics + metatranscriptomics	Active expression of H^+^-ATPases, H^+^/Na^+^ antiporters, ion transport systems (Ca^2+^, K^+^, Mg^2+^, Cl^–^), osmoprotectant pathways (ectoine, proline, trehalose, sucrose), heat-shock proteins (HSP20, DnaK), oxidative stress-response genes and energy metabolism modules (NADH dehydrogenase, cytochrome oxidases) implicated in microbial-mediated salinity adaptation and ion homeostasis.	Microbiome-assisted osmotic adjustment, Na^+^ detoxification, ion homeostasis, stress resilience and enhanced adaptation of mangroves to saline coastal environments.	Lemos-Lucumi et al., 2025
*Arabidopsis thaliana*	*Stutzerimonas stutzeri*, *Chlorella vulgaris*, and their consortium	RNA-Seq transcriptomics	RNA-seq revealed consortium-induced regulation of SOS signaling components (SOS3/CBL4, CIPKs), ion transporters (AKT2, NHX2, CHX17, SKOR), hormone-signaling genes (ABI5, SAURs, EIN3/EIL1), and stress-responsive transcription factors (WRKY25, MYB20, NAC6, NAC042), indicating coordinated regulation of ion homeostasis, osmotic adjustment, hormone signaling, and ROS management under salinity stress. GO enrichment highlighted pathways associated with salt stress response, osmotic stress adaptation, ion homeostasis, ABA-, auxin-, and ethylene-signaling, ROS detoxification, and interspecies interaction processes.	Enhanced biomass accumulation, maintenance of root hair development, improved ion homeostasis and transcriptional reprogramming contributing to salt stress tolerance.	Guendouzi et al., 2025
Wheat (*Triticum aestivum*)	*Tetraploa* sp. E00680	Hybrid genome sequencing (Illumina + Nanopore), transcriptomics, metabolomics, microbiome profiling	Fungal genome (43.51 Mb; 13,096 genes) enriched in secondary metabolism; Genome analysis identified salt-adaptive transporters (*CNH1, NHX1, CTA3, TRK1*, and *KCH1*) and secondary metabolite biosynthetic genes. Transcriptome–metabolome integration revealed activation of the tryptophan–IAA pathway through upregulation of *TAR* and *YUCCA* genes and accumulation of DL-tryptophan, indole-3-lactic acid, and indole-3-acrylic acid. Microbiome profiling showed enrichment of *Bacillus*, *Sphingomonas*, and *Sporosarcina*, associated with phosphorus mobilization and stress adaptation.	Increased K^+^ uptake, reduced Na^+^ accumulation, enhanced phosphorus acquisition and growth under salinity.	Qiu et al., 2026
Dawn redwood (*Metasequoia glyptostroboides*)	*Bacillus paramycoides* JYZ-SD5	Bacterial genomics, host transcriptomics, untargeted metabolomics	Genomic analyses identified genes involved in EPS biosynthesis, compatible solute production (proline, betaine, trehalose), Na^+^/Cl^–^ transport regulation and VOC synthesis. Transcriptomics revealed differential expression of genes associated with nutrient uptake, hormone signaling, ROS detoxification and osmotic regulation, while metabolomics showed activation of ascorbate and proline metabolic pathways.	Enhanced Na^+^ sequestration, osmotic adjustment, antioxidant capacity, nutrient acquisition and root growth under salt stress.	Huang-Fu et al., 2026
*Tamarix* rhizosphere	Native saline rhizosphere microbiome (bacteria and halophilic archaea)	Shotgun metagenomics	Enrichment of Na^+^/H^+^ antiporters (nhaA, nhaB, nhaK, mnh/mrp), V/A-type ATPases, and osmoprotectant-associated genes (*betA/B/C, opuA-D, proA/B/C, ectA-D*); increased genetic information processing, DNA repair, and ion transport functions under high salinity.	Microbial adaptation through enhanced ion homeostasis, osmotic regulation, and stress-maintenance mechanisms; shift from growth-oriented metabolism to cellular homeostasis under saline conditions.	Wang et al., 2026

## Plant transcriptomic regulation under salinity

The RNA-seq approach has contributed immensely to the discovery of genome-scale regulatory networks involved in salinity adaptation, identifying TFs belonging to DREB, WRKY, MYB, NAC, and bZIP families as key regulatory factors (Price et al., 2022; Admas et al., 2025). The transcription factors regulate ion homeostasis, osmolyte accumulation, ABA signaling, and antioxidant defenses. Specifically, many WRKY TFs mediate the SOS pathway and ROS scavenging in response to osmotic stress in various plants (Price et al., 2022). Ion transport and compartmentalization remain vital for salt stress tolerance. Transcriptomic analyses consistently reveal differential expression of key ion-homeostasis genes during salinity stress, particularly members of the SOS, HKT, and NHX families (Wang et al., 2019; Ali et al., 2021; Maach et al., 2024). Functional cooperation between AtSOS1 and AtHKT1 indicates concerted action in ion regulation, whereas mutations in AtSOS1 gene impair the expression of other transporters such as *AtHKT1, AtSKOR*, and *AtAKT1* leading to excessive accumulation of Na^+^ and poor K^+^ uptake (Wang et al., 2019). Analogously, upregulated expression of NHX1 protein leads to increased ion sequestration into the vacuole, suggesting the importance of NHX-mediated compartmentalization in adaptation to salt stress (Ali et al., 2021; Maach et al., 2024). Salt stress also triggers widespread transcriptomic changes in antioxidants. RNA-seq analysis in grapevine (*Vitis vinifera*) showed quick activation of many genes involved in ROS scavenging including *VvAPX5, VvAPX6, VvAPX7, VvAPX8, VvDHAR1, VvMDHAR2*, and *VvGR2* in response to NaCl stress (Liang et al., 2026). Comparison between salt tolerant and sensitive rice genotypes under NaCl treatment using transcriptomics further indicated elevated APX and GR activity in salt tolerant rice cultivars (Fang et al., 2023). In barley too, transcriptome profiling of salt-stressed plants indicates tissue-specific activation of ROS scavenging enzyme genes, such as those encoding peroxidases and enzymes involved in glutathione metabolism, suggesting tissue specificity in antioxidant defense against salinity (Nefissi Ouertani et al., 2021). These host-only studies establish salinity-responsive pathways but do not identify a microbial signal responsible for the observed transcriptional changes. Microbial inoculation altered the host ion-homeostasis gene expression in the presence of salinity, but the mechanistic strength varied among individual instances of experimentation. For instance, Microbial inoculation with *Pseudomonas knacknussii* MLR6 was associated with the increased transcription of SOS1, while *Bacillus megaterium* ZS-3 modified the transcriptomic patterns of NHX1, AVP1, and HKT1, and simultaneous inoculation of *Bacillus atrophaeus* and *Planococcus soli* modified the transcription of genes encoding NHX and HKT transporters (Giannelli et al., 2023). Thus, the experiments identified associations between the microbial treatment and perturbation of the host’s salt-ion-homeostasis mechanisms, but they did not determine the signal of microbial origin responsible for the modifications. In turn, transcriptomic profiling indicates the activation of SOS3 (CBL4), AKT2, and CIPK5 in response to inoculation with *Stutzerimonas stutzeri* and *Chlorella vulgaris* strains (Guendouzi et al., 2025). This provides evidence of a host transcriptional response to inoculation, yet the study does not establish what microbial factor triggered these changes. Experiments with microbial volatiles provide direct links between microbial signals and host responses. For example, the treatment of *Arabidopsis thaliana* plants with 100 mM NaCl led to an increase in the transcript levels of SOS1, SOS2, SOS3, NHX1, NHX2, and APX and a decrease in the expression of HKT1 when exposed to volatiles produced *by B. velezensis* SQR9 compared to controls. This was associated with increased growth, reduced leaf Na^+^ accumulation, and less ROS accumulation. Synthetic 2,3-butanediol reproduced the growth-promoting and salt-protective effects of SQR9 volatiles and was therefore considered to have biological activity in this system. However, its necessity for the complete bacterial VOC response and the requirement of individual host genes were not established (Li et al., 2026). Recent evidence based on the analyses of mutants further underlines that microbial effects with ion homeostasis can involve other channels besides HKT1. Thus, in *A. thaliana* plants exposed to 50 mM NaCl, inoculation with the root-colonizing fungus *Serendipita indica* reduced Na^+^ accumulation in both the parental plants and the hkt1-4 mutants, demonstrating that this effect was independent of HKT1. However, the reduction of Na^+^ accumulation observed upon colonization of wild-type plants was absent in cngc10 and cngc13 mutants, suggesting that CNGCs played a role in the fungal response, although it is not yet clear if their influence on Na^+^ homeostasis was direct or indirect, possibly involving Ca^2+^ (González Ortega-Villaizán et al., 2026). Together, the studies discussed above demonstrate that the regulation of plant ion-homeostasis and antioxidant pathways by microbes is complex and context-dependent, and cannot be simplified to a single conserved transporter or signaling pathway. The SQR9 study shows that microbes can regulate APX and ion-transport genes through volatile-associated processes, while the *S. indica* mutant experiments indicate that lower Na^+^ accumulation can occur independently of HKT1.

The generation of transcriptomic, proteomic, and metabolomic data sets is multi-omics profiling, but not multi-omics integration. The latter implies an explicit analysis of relations between layers, such as, for instance, the comparison of different molecular pathways across layers, or transcript-protein-metabolite associations, or other statistical models. For instance, MOFA+ is a method allowing one to identify sources of variation shared between molecular data sets as well as specific for each layer (Argelaguet et al., 2020). Such approaches may potentially identify relationships between various molecular processes for further validation in experiments, but statistical associations do not prove causal regulation. For example, the multi-omics analysis of *Rosa hybrida* cultivars under 400 mM NaCl demonstrated metabolic alterations specific for particular cultivars (Ren et al., 2024). In this case, the effect of salt stress on *Rosa damascena* Mill. was mainly associated with primary metabolism, while the effect on *R. hybrida* cv. Jardin de Granville was mainly related to secondary metabolism. Additional information was provided through weighted gene co-expression network analysis, which suggested phenylpropanoid and flavonoid biosynthesis as main factors that determine the plants’ resistance to salts. Transcriptomics and proteomics also confirmed the involvement of phenolic acid, lipid, and flavonoid biosynthesis in plant salt tolerance (Ren et al., 2024). Methodological disparities, including primer selection, sequencing strategy, and reference database choice, can substantially influence taxonomic assignment and abundance estimates obtained from microbiome studies. For example, Bacillales have been reported to be overrepresented in some amplicon-based datasets because of primer-related biases (Zheng et al., 2021). Therefore, standardized analytical workflows and integrative sequencing approaches are essential for reliable characterization of salinity-associated microbiomes.

## Microbial metatranscriptomics and rhizosphere activity under salinity stress

Metatranscriptomics have been widely used for elucidating both functions and the role of microbes in salt-affected systems by determining not only their taxonomic diversity but also active processes at the moment of sampling. In contrast to metagenomics, which deals with DNA sequencing data, metatranscriptomics allows us to explore the transcriptionally active metabolic pathways. Thus, we can identify active processes associated with osmoregulation, ion uptake, antioxidative processes, carbon catabolism, and microbial communication in a salinity-stressed environment. The use of this approach provides an opportunity to analyze the metabolically active fraction of rhizosphere microbial communities without the need for assessing microbial abundance through DNA quantification. However, RNA stability and integrity can influence the resulting profile; therefore, RNA preservation, extraction procedures and sample-processing conditions should be considered when comparing metatranscriptomic datasets. Methodological comparisons while examining microbial carbon metabolism in soil slurries, further show that soil RNA extraction procedures differ in yield, integrity, and purity, affecting the material available for transcriptomic analysis (Barber et al., 2024). The use of double-RNA metatranscriptomics in soils exposed to seawater intrusion in paddy rice fields showed an inhibition of methanogenesis together with the reshaping of anaerobic food webs. The transcriptional activity of members of the family Clostridiaceae increased significantly along with the induction of genes involved in the pathways of glycolysis, pyruvate metabolism, ethanol fermentation, acetogenesis, and osmoregulation. Simultaneously, hydrogenotrophic methanogenesis mediated by the family Methanocellaceae decreased, while methylotrophic methanogenesis, promoted by Methanomassiliicoccales and Methanosarcinales, increased during chronic salinity. The transcription levels of osmo-adaptive genes responsible for choline uptake, betaine biosynthesis, flagellin biosynthesis, V/A-type H^+^/Na^+^ -transporting ATPases, and mtaAB methanol utilization gene cluster confirmed substantial reorganization of microorganisms’ metabolism in salt-stress conditions (Zhou et al., 2025). The microbial transcriptional changes observed in soil do not necessarily indicate an equivalent beneficial effect on plant salt tolerance. Drought-stressed rhizosphere metatranscriptomics in Mediterranean pasture ecosystems, too, exhibit extensive changes in taxonomy and function among the microorganisms that help adapt to stress conditions. Microbes belonging to the phylum of Proteobacteria, Bacteroidetes, Burkholderiales, and Rhodospirillales are transcriptionally active in drought conditions, whereas decreases are seen in Actinobacteria. Function-wise, there is considerable activation of genes for oxidative stress response, osmoregulatory signaling, heat-shock responses, and proline synthesis pathways, providing support for the concept of a “rhizospheric meta-organism,” where the plant and its microbiome cooperate to combat environmental stresses (Sharma et al., 2026). Mangrove halophyte metatranscriptomics of *Avicennia germinans* reveal that endophytic microorganisms play an important role in coping with salinity tolerance and stress. The root-attached microbiomes have more transcriptionally active and higher levels of taxonomically diverse communities compared to the leaf-attached communities, with predominant bacteria such as *Pseudomonas*, *Bacillus*, *Streptomyces*, and *Bradyrhizobium*. Genes associated with functions like H^+^-ATPases, H+/Na+ antiporters, efflux pumps, NADH dehydrogenases, cytochrome oxidases, molecular chaperones, heat-shock proteins, ectoine and proline synthesis, trehalose and sucrose metabolism get abundant. Increased expression of genes related to oxidative stress resistance, xenobiotic degradation, hydrocarbon metabolism, and heavy metal detoxification further highlight the diverse roles of endophytic microbiomes under hypersaline environments (Lemos-Lucumi et al., 2025).

From metatranscriptomic studies performed on stressed agricultural soil samples, it is clear that the highly abundant transcripts found are associated with functional microbes contributing to pollutants’ decomposition and metal detoxification. High amounts of transcripts corresponding to enzymes such as catechol 1,2-dioxygenase, gentisate 1,2-dioxygenase, benzoate dioxygenase, mercuric ion reductase, thioredoxin reductase, and selenium metabolic enzymes were observed, along with actively transcribed microorganisms, like those from the *Pseudomonas*, *Achromobacter*, *Bacillus*, *Sphingobium*, *Streptomyces*, *Ralstonia*, and *Arthrobacter* genera. This example illustrates the usefulness of metatranscriptomics for identifying active stress-responsive pathways that help microorganisms adapt to their environment and detoxify themselves (Sharma and Sharma, 2018). Not only are metatranscriptomics capable of resolving such functional pathways and activities in natural environments but also when metatranscriptomics and metagenomics are combined, active pathways within fermented food microbiomes can be identified as well. Fermented bamboo shoots showed differences in *Lactiplantibacillus plantarum* strains’ transcriptional regulation of pyruvate metabolism, lactose consumption, aromatic amino acid biosynthesis, and carbohydrate-active enzymes’ modules, highlighting the identification of metabolically important microbes and their functions through transcriptional profiling (Wu et al., 2025).

Together, these series of studies clearly show that metatranscriptomics provides a strong systems-based approach to understand the process of microbial adaptation to salinity and other environmental stressors through the coupling of active community structure with functional expression. The recurrent transcription factors identified in relation to stress adaptations include osmoprotectants production, regulation of ion homeostasis, antioxidant mechanisms, carbohydrate metabolisms, biofilm formation, modification of methanogenesis, and plant-microbe signaling pathways.

## Proteomic reprogramming in salt stress

Salt stress effect on plants results in significant changes in the proteome of plants, which enable them to sustain ion equilibrium, osmotic pressure, redox status, and metabolic balance despite exposure to salty environments. According to comparative studies carried out by 2-DE, LC–MS/MS, and quantitative proteomics, salinity responsive proteins are related to such biochemical pathways as photosynthesis, antioxidative responses, osmolyte synthesis, protein folding, signal transduction, and ion transport (Arefian et al., 2019; Raza et al., 2023; Usman et al., 2025). Comparison between proteomic patterns in salt tolerant and salt sensitive genotypes proves that salt tolerant plants maintain faster and more pronounced induction of the aforementioned proteins. As for 2-DE analysis in chickpeas (*Cicer arietinum*), it revealed differential regulation of 364 protein spots under NaCl exposure and proved greater intensity of expression of proteins associated with photosynthesis, stress responses, and protein synthesis in tolerant genotype (Flip 97-43c) as compared with the other (susceptible) genotype (Flip 97-196c) (Arefian et al., 2019). Chlorophyll-related proteins, ATP synthase proteins, RuBisCO proteins, oxygen evolving enhancer complex proteins, fructose-bisphosphate aldolase, carbonic anhydrase, elongation factor Tu, LEA-2 proteins, and heat shock proteins proved especially numerous in tolerant genotype and thus demonstrated proteomic acclimatization to stress conditions caused by salinity (Arefian et al., 2019). Proteomic measurements are limited by the analytical sensitivity and the depth of sampling of the peptide space. Because in data-dependent LC–MS/MS analyses only a small fraction of identified features is typically selected for fragmentation, and sensitivity is often limited, especially for low-abundance proteins (Michalski et al., 2011). Therefore, the absence of a protein in an untargeted proteomic dataset should not be used as definitive evidence.

In line with multiple transcriptomic data, the proteomics work further substantiate the importance of Proteomic studies further corroborate transcriptomic findings by demonstrating increased abundance or activation of proteins associated with ion homeostasis, including SOS pathway components, HKT transporters, NHX antiporters, MAPKs, and Ca^2+^-dependent signaling proteins under salinity stress (Figure 3). A phosphoproteomic study using iTRAQ technology for cucumbers (*Cucumis sativus*) showed that the application of spermidine induced higher phosphorylation levels in response to saline conditions such as SOS2 kinase, SOS3 calcium-binding protein, and other stress-related regulators (Li et al., 2025). Proteomic studies have characterized several salinity-induced protein components such as NHX Na^+^/H^+^ antiporter, HKT transporter, V-type H^+^-ATPase, SOS2–CBL proteins, MAPKs, and aquaporins involved in Na^+^ compartmentalization and K^+^/Na^+^ balance (Athar et al., 2022). Furthermore, integration of proteomics and omics data suggests that salt tolerance is highly related to the integrated regulation of LEA proteins, ABA-signaling, ion homeostasis and antioxidant defense pathways. Several proteins like LEA14, HKT transporters, Na^+^/H^+^ antiporter, ROS detoxification enzymes and ABA-regulated proteins play essential roles in maintaining osmotic balance, ionic homeostasis, and cellular integrity under NaCl stress. Integrated omics approach indicates extensive cross-talk between proteome remodeling, transcriptional regulation, and metabolic adaptation during salt tolerance process (Hao et al., 2025). Salt stress conditions also lead to differential accumulation of membrane proteins involved in ionic transport, vesicle transport, ATPase activity, and signaling processes in soybean. Analysis have shown that expression levels of various membrane proteins increase such as V-type proton ATPase subunit A/E1/E3, ABC transporters (ABCC2, ABCG8), Ammonium transporter AMT1;2, Ras-related protein Rab-1A, Annexin D1/D2, Cytochrome c oxidase subunits, NAD(P)H dehydrogenases, DNAJ chaperone family, receptor kinases, and proton pump interactors during NaCl stress. KEGG pathway analysis indicates upregulation of endocytosis, SNARE-mediated vesicular transport, fatty acid metabolism, carbon metabolism, and amino acids synthesis pathway upon salt acclimation (Rehman et al., 2022). An example of a host-mediated stress response mechanism is the wheat TaHSP17.4–TaHOP interaction wherein an analysis of the interaction, together with gene overexpression and stress phenotyping, established its involvement in plant stress tolerance (Wang Y. X. et al., 2023). However, since the researchers did not investigate microbial inoculation or activity, the work establishes a link between the host stress response and salt tolerance but does not establish its mediation by microbes.

Salinity-induced proteomic remodeling occurs within the context of adaptive strategies for coping with the environmental stresses induced by the presence of salt ions in rhizospheres. Previous reports have shown that salt stress results in alteration of root exudation profiles, favoring rhizosphere communities enriched with halotolerant microbes, such as *Sphingomonas, Streptomyces, Bacillus*, and *Pseudomonas*, which play a role in the regulation of ions, osmoprotectant production, antioxidant activation, and hormone signaling in salt-stressed plants (Xu et al., 2025; Cheng et al., 2025; Table 2). A recent work highlighted the role of rhizosphere function in supporting stress-responsive reprogramming of proteome and metabolism in salt-stressed crops due to microbial exopolysaccharide production, VOC generation, ACC deaminase activity, and compatible solute production (Ren Y. et al., 2025). Moreover, studies suggest that PGPMs, such as *B. subtilis*, display increased biofilm formation and antimicrobial compound production after root colonization mediated by the stimulation caused by root carbohydrates, such as xylan. During drought and/or salinity-associated stress, the inoculation of PGPM results in enhanced levels of antioxidants, such as superoxide dismutases and peroxidases, thus leading to improved ROS scavenging capacity and osmotic regulation in the host plant. The elevated activity of ACC deaminase is observed in stress-tolerant strains, with *Pseudomonas* sp. Y1 exhibiting enzyme activity level of 895 ± 35 nmol α-ketobutyrate mg^–1^ protein h^–1^ (Mukherjee, 2022).

**TABLE 2:** The effect of halotolerant microorganisms on the biochemical and cellular responses of plants to salinity stress.

Microorganism	Principal mechanisms	Salinity mitigation effects	Reported NaCl tolerance (mM)	Major PGP traits	References
*Azotobacter chroococcum* NBRC + *Alcaligenes faecalis* NBRC13111	Enhanced ROS scavenging, osmotic adjustment, ion homeostasis	Shoot length (+64.6%), root length (+69.8%), shoot DW (+282.8%), reduced Na^+^ accumulation	–	Antioxidant induction, osmolyte accumulation, improved K^+^ uptake	Abdel Latef et al., 2021
*Enterobacter cloacae* PM23	Ethylene reduction, nutrient acquisition, ROS detoxification	Biomass (+46%–58%), chlorophyll (+14%–30%), lower MDA, H_2_O_2_ and electrolyte leakage	300–900	ACC deaminase, IAA, EPS, siderophores	Ali et al., 2022
*Stenotrophomonas nematodiphila* BZH3_2	Improved Na^+^/K^+^ homeostasis, enhanced photosynthesis	Aboveground FW (+107.9%), root length (+58%), increased osmolytes	500–2,500	Biochar-assisted colonization, improved N availability	Cheng et al., 2025
*Glutamicibacter* sp.	Nutrient cycling, osmotic adjustment, ROS detoxification	Shoot DW (+49%), root DW (+29%), reduced Na^+^ and Cl^–^ accumulation	600	IAA production, antioxidant stimulation, enhanced soil enzymes	Hidri et al., 2022
*Pseudomonas* sp.	Improved nutrient uptake, ion balance, membrane protection	Shoot/root DW ratio (+61%), decreased MDA and electrolyte leakage	600	ACC deaminase, EPS, IAA	Hidri et al., 2022
*Solibacillus isronensis* CR2	Nutrient mobilization and phytohormone regulation	Enhanced wheat growth and yield under salinity	855	IAA, phosphate solubilization, siderophores, HCN	Radhakrishnan and Krishnasamy, 2024
*Bacillus xiamenensis* ASN-1 + SNP	Antioxidant activation, osmotic regulation, stress signaling	Shoot DW (+196%), root DW (+247%), reduced Na^+^ and MDA	2,500	IAA, ACC deaminase, NO signaling	Sharma et al., 2021
PGPR PH2970 + PH3000	Improved photosynthesis, osmotic adjustment, ion homeostasis	Photosynthesis (+60.8%), yield (+17%), Na^+^ reduced by 45.7%	38	Carbohydrate metabolism activation, antioxidant enhancement	Xu et al., 2025
*Bacillus aryabhattai* H19-1	ABA-independent antioxidant pathway	Shoot DW (+20%), root DW (+25%), improved chlorophyll retention	–	Increased chlorophyll, carotenoids and Ca^2+^	Yoo et al., 2019
*Bacillus mesonae* H20-5	ABA-dependent osmotic adjustment	Shoot DW (+19%), total biomass (+21%), enhanced chlorophyll stability	–	ABA induction, proline accumulation, antioxidant activation	Yoo et al., 2019
*Halolamina pelagica* CDK2	Nutrient mobilization and osmoprotection	Enhanced chlorophyll, root growth and stress-responsive gene expression	1,300	IAA production, P/K/Zn solubilization, trehalose synthesis	Naitam et al., 2023
PGPR consortium (*Vibrio spartinae* HPJ2, *Pseudarthrobacter oxydans* SRT15, *Bacillus zhangzhouensis* HPJ40)	Enhanced photosynthesis, antioxidant capacity and ion homeostasis	Biomass (4.5-fold), iWUE (>10-fold), shoot Cu reduced by ∼50% in HPJ2 + HPJ40 consortium	171–1,027	ACC deaminase, IAA, improved nutrient uptake	Mesa-Marín et al., 2026
*Bacillus safensis* AL	Improved ion homeostasis and membrane stability	Grain yield (+43%), stem DW (+52%), proline (+50%)	1,600	Osmolyte accumulation, nutrient mobilization	Amini Hajiabadi et al., 2021
*Bacillus pumilus* HR	Osmotic protection and chlorophyll stabilization	Proline (+50%), phenolics (+45%), improved grain quality	1,600	ACC deaminase, osmolyte and antioxidant enhancement	Amini Hajiabadi et al., 2021
*Zhihengliuella halotolerans* SB	ROS scavenging, osmotic balance and ion transport	Soluble sugars (+34%), proline (+31%), improved K^+^/Na^+^ ratio	–	Proline, soluble sugars and nutrient accumulation	Amini Hajiabadi et al., 2021

## Systems biology approaches for microbe-assisted salt tolerance

System biological tools have been shown to be an effective strategy for investigating the sophisticated molecular events behind microbe-mediated salt tolerance in plants. Systems biology integrates information generated from genomics, transcriptomics, proteomics, metabolomics and computational modeling to elucidate complex plant–microbe interactions under salinity stress. Furthermore, multi-omics analysis methods have significantly contributed to knowledge about plant-microbe interaction in salinity-stressed plants (Table 1). Hybrid Illumina and Nanopore sequencing of a salt-tolerant fungal endophyte, *Tetraploa* sp. E00680, from wheat (*Triticum aestivum*) identified 43.51 Mb genome containing 13,096 annotated genes with high abundance in secondary metabolism and microbial metabolism pathways. Comprehensive analysis of transcriptomics, metabolomics, and rhizospheric microbiome with systems biology/network modeling/approaches indicated that inoculation of E00680 can increase K^+^ content, reduce Na^+^ content, improve phosphorus content, and trigger tryptophan biosynthesis involved in auxin synthesis under salt stress (Qiu et al., 2026). Likewise, in *M. glyptostroboides*, genome analysis of PGPR *Bacillus paramycoides* JYZ-SD5, together with host transcriptomics and untargeted metabolomics, provided insights into PGPR-mediated induction of reactive oxygen species scavenging, osmoregulation, nutrient acquisition, and hormonal-related pathways for salt adaptation. The genome of this bacterium contained genes associated with exopolysaccharide synthesis, osmolyte biosynthesis, ion homeostasis, and volatile organic compound biosynthesis. Bacterial inoculation under salt stress also increased ascorbic acid and proline synthesis pathways in plants (Huang-Fu et al., 2026). Metabolite annotation is a challenge in untargeted microbial metabolomics as existing spectral libraries rarely cover the full range of microbial metabolites, and are skewed toward analytes for which reference standards are available commercially or easily accessible (Zuffa et al., 2024). Similarly, in licorice (*Glycyrrhiza uralensis*), integrated metabolomics and transcriptomics analysis showed that inoculation with *B. subtilis* leads to arginine/proline metabolism, phenylpropanoid metabolism, antioxidant metabolism, and cell wall-related gene regulation, such as Xyloglucan Endotransglucosylase (XET), GPX, and Caffeic Acid O-Methyltransferase (COMT) during PGPR-mediated salinity adaptation (Xiao et al., 2024). Metabolite annotation in non-targeted metabolomics remains challenging due to incomplete spectral-library coverage and the difficulty of accurately predicting fragmentation patterns, especially for the chemically diverse metabolites produced by microorganisms (Vitale et al., 2025).

Furthermore, network biology and computational modeling techniques have facilitated a comprehensive systems-level understanding of the regulation of stress response mechanisms via interactions. Based on transcript-metabolite correlations, microbiome modeling, and temporal clustering analysis, several salinity-responsive modules, associated with flavonoid biosynthesis, amino acid metabolism, ROS detoxification, ABA signaling, NHX antiporters, HKT transporters, SOS signaling, and osmolyte biosynthesis, have been reported. The application of proteogenomic machine learning that incorporates data from both transcriptomics and proteomics, it was found that adding interactions from STRING and CORUM databases significantly increased the accuracy of predictions for over 13,000 proteins, as opposed to cognate transcript models, highlighting the presence of considerable post-transcriptional interaction-dependent regulation in response to stress (Srivastava et al., 2022). A variety of systems biology strategies have been developed for designing stress-resistant phyto-microbiomes in salty conditions. Specifically, the implementation of artificial microbial communities, CRISPR/Cas genome editing, host-based microbiome manipulation, and microbiome interaction modeling is being used to modulate rhizosphere signaling, nutrient cycles, ion homeostasis, and recruitment of stress-resistant microbes to enhance crop resistance to salinity (Patyal et al., 2025). Computational modeling approaches provide complementary methods for predicting plant-microbe interactions, but differ in their inputs and biological outcome predictions. Genome-scale metabolic (GEM) models use reactions encoded by the genome to simulate the production, consumption and interdependencies of metabolites under given environmental constraints. For instance, modeling of 193 Arabidopsis root bacterial genomes predicted environment-dependent metabolic dependencies and putative functional redundancies in synthetic communities (Mataigne et al., 2022). Genome-informed trait-based models focus on predicting ecologically-relevant strategies and resource use traits, using genome-derived properties to inform microbial growth, resource competition and environmental responses in changing rhizosphere conditions (Marschmann et al., 2024). Metagenomic association approaches instead seek to statistically associate microbial taxa or community properties with host phenotype measurements, rather than directly predicting microbial metabolism. In soybean, this approach identified associations between microbial taxa and a salt tolerance index, which were followed up with inoculation experiments identifying *Thalassospira xiamenensis* as a candidate salt tolerance-promoting bacterium under NaCl stress (Luo et al., 2026). Thus, GEM models predict metabolic interdependencies, trait-based models predict ecological resource strategies, and metagenomic association approaches identify candidate microbe-host relationships for further experimentation. The predictions should not be taken as established field-level salt tolerance models, but could be advanced by future studies combining modeling and longitudinal multi-omics with measured environmental conditions, including metabolite exchanges, microbial growth rates, and plant physiology, followed by independent validation in controlled and field salinity conditions.

Evidence of microbial salt tolerance in laboratory studies does not guarantee efficacy in saline field conditions. Survival and establishment after application can be affected by factors in the soil, the environment, and the formulation. In pot experiments with different NaCl concentrations and temperatures, encapsulated *B. subtilis* showed better survival than free cells, including in nutrient-poor, acidic clay soil (Graciano et al., 2025). This suggests that the survival of inoculant in the targeted soil and environment should be evaluated. Another factor to consider is competition with indigenous microbiota. A study found that free *Pantoea alhagi* NX-11 survived less well in non-sterile than in sterilized soil, which might reflect suppression by indigenous microbiota, although the interactions were not identified (Yang et al., 2026). Encapsulation improved survival during storage and colonization of roots and was associated with a 15.8% increase in rice grain yield compared to free-cell inoculation in a saline field trial. Host compatibility is also an issue. In greenhouse experiments with barley, AMF-enriched native microbial inocula had largely negative or neutral biomass responses to salinity, while host genotype and inoculum identity influenced microbial community assembly (Royle et al., 2026). Isolation from a saline environment does not establish compatibility per se and beneficial effects in a target crop. Inoculant and host combinations should therefore be tested in field experiments using a range of host genotypes, locations, and growing seasons in non-sterile saline soils. Electrical conductivity, pH, nutrient status, moisture, and temperature in the soil should be recorded, and trials should include uninoculated and carrier controls. The number of viable inoculum should be determined at the time of application, and establishment and persistence should be monitored alongside crop yield, tissue Na^+^/K^+^ balance, and indicators of salt injury.

Overall, these findings show that the salinity-induced changes in the microbiome involve taxonomical, functional, and molecular modifications that contribute to plant resilience to high salinity levels. It can be assumed that the selection pressure imposed on fungal populations in terms of their colonization abilities, metabolic plasticity, and stress responses results in the proliferation of taxa able to foster the plant growth through coordinated management of ion balance, osmotic adjustments, oxidative defense, and interactions within the rhizosphere. Summing up, it is safe to conclude that salinity tolerance of plants is based on the coordination of several molecular and ecological processes, including ion homeostasis, antioxidant activity, metabolic changes, and stress adaptation mediated by microbiomes.

## Conclusion and future directions

In agriculture, soil salinity is still one of the primary threats to overall production, with negative impacts on plant growth, productivity, and ecosystem resilience. Beneficial microorganisms have also emerged as effective biological allies to increase plant tolerance to salinity by multiple mechanisms, such as nutrient absorption, ion homeostasis, osmotic control, phytohormone regulation, and reinforcement of antioxidant defense. Microbial-mediated promotion of both enzymatic and non-enzymatic antioxidants is an important factor in the alleviation of oxidative stress and maintenance of cellular integrity in saline environments, among other mechanisms. Recent years have seen great progress in omics technologies which improved our knowledge base about the intricate molecular interactions at play in plant-microbe relationships under salinity stress. Genomics, transcriptomics, proteomics, metabolomics, and metagenomics have all brought to light important genes, proteins, metabolites, signaling networks, and microbial communities of plant adaptation under stress. Integrating these omics approaches can provide complementary insights into microbial community composition, functional potential and activity, host responses, metabolic changes, and related stress phenotypes. However, the strength of the resulting mechanistic interpretation depends on how well the different data layers can be integrated and supported by independent validation. Standardized multi-omics protocols should use coordinated sampling across data layers, consistent reporting of salinity conditions and sample metadata, clearly documented quality-control and normalization procedures, and integration methods that are appropriate for the biological question being addressed. Translating laboratory findings to field applications can be limited by environmental variables, competition with indigenous microbiota, host specialization, inoculant establishment, and persistence, and physicochemical complexity of natural soils. Thus, field trials should involve replicate experiments in non-sterile saline soils with varying salinity levels, host genotypes, locations, and seasons. The soil salt content, pH, irrigation regimes, and other environmental factors that could impact inoculated plants should be measured, and appropriate uninoculated and carrier controls should be included. In addition, plant growth and yield, inoculant establishment and persistence, plant ion status, and salt damage should be evaluated. Future studies should combine paired host and microbial datasets by using cross-omics pathway or network analyses, identify molecular associations that converge on plant ion homeostasis, oxidative damage, and growth responses, and experimentally validate these putative mechanisms to discriminate between causal and correlative relationships. Synthetic microbial consortia, precision microbial inoculants, and microbiome-assisted breeding approaches are all new promising approaches for crop improvements in saline soils.

## Footnotes

1
https://www.fao.org/newsroom/detail/fao-launches-first-major-global-assessment-of-salt-affected-soils-in-50-years/en
